# Identification of CBPA as a New Inhibitor of PD-1/PD-L1 Interaction

**DOI:** 10.3390/ijms24043971

**Published:** 2023-02-16

**Authors:** Fengling Wang, Wenling Ye, Yongxing He, Haiyang Zhong, Yongchang Zhu, Jianting Han, Xiaoqing Gong, Yanan Tian, Yuwei Wang, Shuang Wang, Shaoping Ji, Huanxiang Liu, Xiaojun Yao

**Affiliations:** 1 College of Chemistry and Chemical Engineering, Lanzhou University, Lanzhou 730000, China; 2 Henan International Joint Laboratory for Nuclear Protein Regulation, Cell Signal Transduction Laboratory, School of Basic Medical Sciences, Henan University, Kaifeng 475004, China; 3 Ministry of Education Key Laboratory of Cell Activities and Stress Adaptations, School of Life Sciences, Lanzhou University, Lanzhou 730000, China; 4 Faculty of Applied Science, Macao Polytechnic University, Macao 999078, China; 5 College of Pharmacy, Shaanxi University of Chinese Medicine, Xianyang 712046, China; 6 State Key Laboratory of Quality Research in Chinese Medicine, Macau Institute for Applied Research in Medicine and Health, Macau University of Science and Technology, Macau 999078, China

**Keywords:** PD-1, PD-L1, small-molecule inhibitor, CBPA, cancer immunotherapy

## Abstract

Targeting of the PD-1/PD-L1 immunologic checkpoint is believed to have provided a real breakthrough in the field of cancer therapy in recent years. Due to the intrinsic limitations of antibodies, the discovery of small-molecule inhibitors blocking PD-1/PD-L1 interaction has gradually opened valuable new avenues in the past decades. In an effort to discover new PD-L1 small molecular inhibitors, we carried out a structure-based virtual screening strategy to rapidly identify the candidate compounds. Ultimately, CBPA was identified as a PD-L1 inhibitor with a K_D_ value at the micromolar level. It exhibited effective PD-1/PD-L1 blocking activity and T-cell-reinvigoration potency in cell-based assays. CBPA could dose-dependently elevate secretion levels of IFN-γ and TNF-α in primary CD4^+^ T cells in vitro. Notably, CBPA exhibited significant in vivo antitumor efficacy in two different mouse tumor models (a MC38 colon adenocarcinoma model and a melanoma B16F10 tumor model) without the induction of observable liver or renal toxicity. Moreover, analyses of the CBPA-treated mice further showed remarkably increased levels of tumor-infiltrating CD4^+^ and CD8^+^ T cells and cytokine secretion in the tumor microenvironment. A molecular docking study suggested that CBPA embedded relatively well into the hydrophobic cleft formed by dimeric PD-L1, occluding the PD-1 interaction surface of PD-L1. This study suggests that CBPA could work as a hit compound for the further design of potent inhibitors targeting the PD-1/PD-L1 pathway in cancer immunotherapy.

## 1. Introduction

T-cell-mediated cellular immunity is strictly supervised by a check/balance system functioning through co-stimulatory and inhibitory signals (i.e., immune checkpoints) [1,2]. Once cancer develops, the balance between these positive and negative signals is tipped toward immunosuppression [3]. Therefore, relieving the negative checkpoints for the antitumor immunity and/or enhancing the activation signals of the tumor-infiltrating effector T cells (Teffs) represent potentially appealing avenues for cancer therapy [4]. In this context, the programmed death 1 (PD-1) checkpoint/programmed death-ligand 1 (PD-L1) is one of the most promising targets for immunotherapeutic purposes [5,6].

PD-L1, the ligand of PD-1, is expressed on the surface of various solid tumors [7,8,9], and its aberrantly high expression in tumor cells or presence in the tumor microenvironment (TME) can make tumors more susceptible to PD-1/PD-L1 inhibitors. PD-1/PD-L1 inhibitors can block PD-1′s binding to PD-L1, interrupt the negative regulatory signals, and reinvigorate the activity of effector T cells, thus strengthening the antitumor immune response [5]. Since 2014, the US FDA has approved six PD-1/PD-L1 inhibitors for cancer treatment, all of them being monoclonal antibody drugs, including pembrolizumab, nivolumab, duvalumab, atezolizumab, avelumab, and cemiplimab. These monoclonal antibodies have achieved great clinical success in the treatment of a variety of malignancies, including melanoma, bladder cancer, metastatic non-small-cell lung cancer, and skin Merkel cell carcinoma [10,11,12]. However, monoclonal antibodies are associated with several disadvantages, such as high production costs, immunogenicity, lower bioavailability, the poor permeability of solid tumor tissue, and difficult controlled pharmacokinetics, thereby hindering their antitumor efficacy [13,14,15]. An increasing amount of research is devoted to blocking the PD-1/PD-L1 interaction by applying small-molecule compounds to address these drawbacks of antibody drugs, opening a new avenue for tumor immunotherapy. 

Compared with antibodies, small-molecule inhibitors possess several advantages, such as low manufacturing costs and low immunogenicity, high stability, reasonable half-life, and high tissue and tumor penetration [16,17,18]. A series of patents from the BMS company disclosed a class of biphenyl-type small-molecule inhibitors with good blockade activity against PD-1/PD-L1 binding [19,20]. It has been reported by the Holak group that such small-molecule compounds act directly upon the surface of the PD-L1 protein, inducing in PD-L1 dimerization; for example, BMS-202 is sandwiched between two dimerized PD-L1 cylinders in the shape of a hydrophobic cavity [21,22]. These BMS inhibitors displayed good biochemical and cellular activity, while efficacy and safety evaluations for them in vivo are yet to be undertaken. In addition to the BMS inhibitors, Feng’s research group disclosed a class of bromobenzyl ether derivatives that can potently block the interaction of PD-1 and PD-L1 at the biochemical level, and the sodium salt form of the test compound at a dose of 15 mg/kg displayed obvious antitumor activity in a mouse B16F10 subcutaneous xenograft model. The scaffold of this kind of compound is similar to that of the BMS inhibitors, while the difference is the replacement of the methyl group in the biphenyl structure with bromo [23,24,25]. MAX-10181, an orally active PD-L1 inhibitor from Maxinovel Pharmaceuticals demonstrating similar efficacy as the PD-L1 antibody durvalumab, is currently undergoing phase I clinical trials (NCT04122339) in Australia. To date, a large number of companies and research institutions have devoted research to the development of novel small-molecule agents targeting PD-1/PD-L1, but the vast majority of small molecules are still undergoing preclinical studies [26,27,28,29]. More comprehensive factors, addressing the biosafety, pharmacokinetic, and pharmacodynamic properties of these reported inhibitors, need to be further explored. Moreover, it is important to identify and design novel small-molecule inhibitors that possess diverse pharmacodynamic and pharmacokinetic properties and verified antitumor immune responses. Rational combinations of these small-molecule inhibitors with conventional monoclonal antibodies might contribute to greater therapeutic benefits that those of either agent as a monotherapy and/or overcome drug resistance-related mechanisms [30].

In this article, we report the discovery of a new PD-L1 inhibitor, N-{4-[(4-chlorobenzyl)oxy]benzyl}N-(4-pyridinylmethyl)amine (CBPA), that can interrupt the interaction of PD-1 and PD-L1 at the micromolar level. CBPA exhibited significant in vivo antitumor efficacy in primary tumor models and simultaneously increased tumor-infiltrating CD8^+^ T cells and cytokine secretion in the TME. Thus, CBPA could serve as a potential hit compound for immunotherapeutic drug discovery.

## 2. Results

### 2.1. Ligand and Structure-Based Virtual Screening

To discover potential PD-L1 inhibitors, ligand and structure-based virtual screening was carried out with approximately 200,000 compounds in the Specs database. To evaluate the virtual screening capabilities of the different crystal structures, a cross-docking study was performed. As revealed in Supplementary Table S1, the docking capabilities of the four crystal structures (5J8O, 5J89, 5N2D, and 5N2F) were diverse. Overall, when the root-mean-square deviation (RMSD) value was no more than 2 Å, no crystal structure was able to perfectly predict the binding conformations for all the co-crystallized ligands. Among these four crystal structures, 5J89 and 5J8O could reproduce the binding conformations for the ligands with both SP and XP precision. Further, 5J89 could reproduce the binding conformations for two ligands, while 5J8O could only reproduce the binding conformations for one ligand with SP precision. Then, the ROC curves and AUC values were employed to evaluate the screening powers of the crystal structures 5J89 and 5J8O. The AUC values of the crystal structures 5J89 and 5J8O were 0.85 and 0.80, respectively, using Glide SP and 0.95 and 0.98, respectively, using Glide XP (Supplementary Figure S1). Therefore, 5J89 was finally selected as the initial structure for the final virtual screening. The docking score for the co-crystal ligand (BMS-202) was −10.144 kcal/mol when it was redocked into the original binding site of the crystal structure 5J89 with SP precision (Supplementary Figure S2).

**Figure 1:**
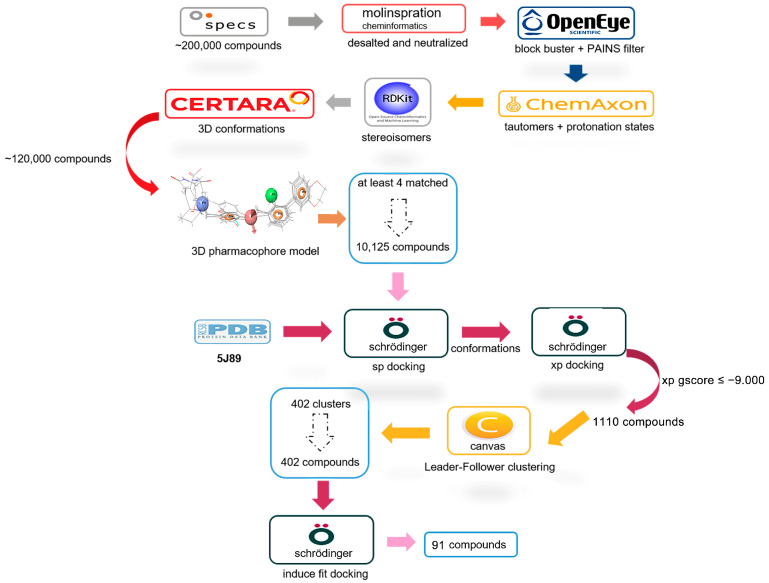
The flowchart for the structure-based virtual screening.

The workflow for the virtual screening analysis is shown in Figure 1. All compounds in the Specs database were first preprocessed according to a series of filtering criteria; a total of 120,000 compounds were filtered out and their property distributions are shown in Supplementary Figure S3. Then, these compounds were screened with preliminary pharmacophore-based virtual screening. Three-dimensional (3D) pharmacophore models based on the structures of 5J89 and multiple ligands are shown in Supplementary Figure S4. Next, through two-level (SP and XP) screenings, 1110 top-ranked compounds with docking XP Gscores ≤ −9.000 kcal/mol were obtained. Furthermore, in order to remove redundancy and ensure the structural diversity of the selected hits, we used the clustering protocol in the Canvas module to cluster these molecules into 402 groups with Tanimoto coefficients of less than 0.5. The compounds with the best scores in each group were retained and subjected to fully flexible docking using the Induced Fit module. Finally, a total of 91 chemicals were selected and purchased for subsequent bioactivity validation. Based on the experimental tests, we found that compound N-{4-[(4-chlorobenzyl)oxy]benzyl}-N-(4-pyridinylmethyl)amine (SPECS No. AN-465/42833793, CBPA) exhibited bioactivity at the molecular and cellular levels. 

### 2.2. Binding Affinity of CBPA to PD-L1

**Figure 2:**
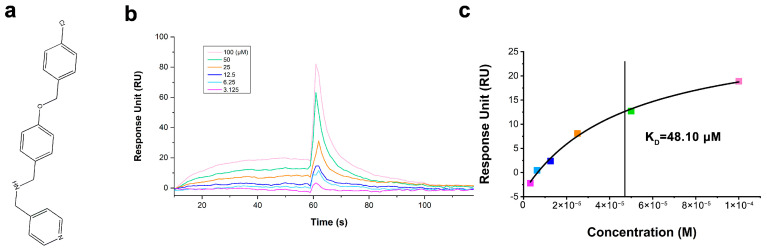
The binding affinity of the compound CBPA with hPD-L1 was measured with SPR. (**a**) The chemical structure of CBPA. (**b**) Overlay of sensorgrams for the interactions between hPD-L1 and CBPA. (**c**) Response data at equilibrium versus CBPA concentration.

The interaction between CBPA and hPD-L1 was evaluated with the SPR using a Biacore X100 instrument. Recombinant hPD-L1 ectodomain (residues 18–239) was selected for immobilization onto a CM5 sensor chip. CBPA at a wide range of half-diluted concentrations (from 3.13 to 100 μM) was guided over the sensor chip surface. The binding responses were recorded as sensorgrams. A single-site model was used to fit the obtained equilibrium binding responses versus ligand concentrations. Our previous study showed that the K_D_ value of inhibitor 1 (BMS-1) for binding to hPD-L1 was 3.16 μM [31], providing a positive reference to confirm that the immobilized hPD-L1 was functional. As illustrated in Figure 2, an increasing concentration of CBPA led to an increasing response unit value, and the K_D_ value of CBPA binding with hPD-L1 was calculated to be 48.10 μM. This observation indicated that the compound CBPA could be an active hPD-L1-binding molecule with determinable affinities. 

### 2.3. Blockade of the PD-1/PD-L1 Interaction with CBPA at the Cellular Level

**Figure 3:**
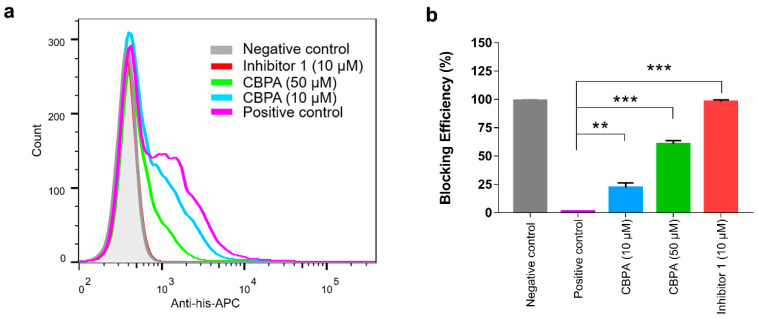
Inhibitory activity of CBPA based on flow cytometry assays. (**a**) Representative flow cytometry histogram for evaluating the efficacy of CBPA in disrupting hPD-L1–hPD-1 interaction. (**b**) Blocking efficiencies are displayed as means ± SEM from three separate experiments, ** *p* < 0.01, *** *p* < 0.001, unpaired *t*-test.

In order to evaluate whether CBPA could interrupt the PD-1/PD-L1 protein–protein interaction (PPI) at the cellular level, Jurkat cells stably overexpressing hPD-1-EGFP were established. His-tagged hPD-L1 recombinant protein with a constant concentration was incubated with these hPD-1-Jurkat cells, with or without the compound CBPA at various concentrations (10 or 50 μM). Binding of the dissociative PD-L1 protein to PD-1 on the cell membrane of Jurkat cells was detected with APC-labeled anti-His antibody and was analyzed with flow cytometry. As indicated in Figure 3a, no positive cells were detected in the negative group on account of the lack of His-tagged PD-L1. Conversely, the positive control incubated under the CBPA-free condition exhibited the greatest average fluorescence intensity signal, which confirmed that the staining was specific. In addition, we determined the blocking efficiency of inhibitor 1, using a reference standard to certify the rationality of the experiment system. As illustrated in Figure 3, CBPA could significantly decrease PD-L1 binding to the surface of Jurkat cells. The blocking efficiency for CBPA against PD-1/PD-L1 PPI at a 10 μM concentration was 22.15%, whereas, with the presence of CBPA at a 50 μM concentration, the blocking efficiency was increased to 60.67% (Figure 3b). Moreover, during the flow cytometric experiments, toxicity was not observed for the Jurkat cells in the presence of CBPA at a 50 μM concentration, confirming that CBPA possesses potency for binding PD-L1 and blocking cell-surface PD-1/PD-L1 interaction.

### 2.4. Immunoregulatory Activity Based on Luciferase Reporter Assay

**Figure 4:**
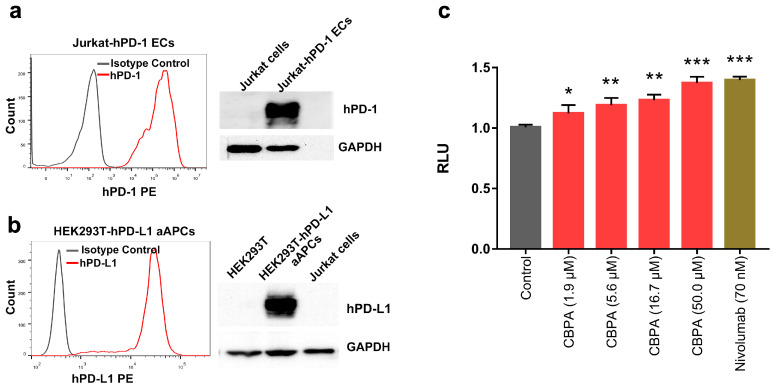
Activity of CBPA in PD-1/PD-L1 checkpoint assay. (**a**,**b**) Western blot and flow cytometry analysis of the expression of the hPD-1 protein in the Jurkat-hPD-1 effector cells and the expression of the hPD-L1 protein in the HEK293T-hPD-L1 aAPCs, respectively. (**c**) PD-1/PD-L1 checkpoint luciferase reporter assay was used to examine the effects of CBPA in restoring T-cell function. Relative luminescence is normalized to the cells-only groups. Data are displayed as means ± SEM, * *p* < 0.05, ** *p* < 0.01, *** *p* < 0.001, unpaired *t*-test.

The compound CBPA was also tested in a luciferase reporter PD-1/PD-L1 blockade assay (Figure 4). In this assay, the effector Jurkat cells were engineered to constitutively express hPD-1 and contained a luciferase reporter gene under the control of the TCR-inducible NFAT promoter (hPD-1 effector cells (ECs)). When these cells were co-cultured with the antigen-presenting surrogate HEK 293T cells, which constitutively express the TCR agonist and hPD-L1 (hPD-L1 aAPCs), TCR signaling was repressed by the PD-1/PD-L1 interaction. With the addition of the inhibitors of the PD-1/PD-L1 complex, EC activation was restored, as shown by an increased luminescence intensity. As shown in Figure 4a,b, Western blot results confirmed that ECs overexpressed PD-1 and aAPCs overexpressed PD-L1, while wild-type Jurkat cells and HEK 293T cells showed no obvious expression of these two proteins. Moreover, the hPD-1 and hPD-L1 proteins were successfully expressed on the surface of the ECs and aAPCs, respectively, as verified by the flow cytometry analysis. As revealed in Figure 4c, nivolumab was used as the positive control and induced significantly higher luminescence intensity in treated ECs compared to the “cells-only” negative control. Additionally, CBPA could dose-dependently improve the activity of the TCR signaling, and the addition of CBPA at a 50 μM concentration increased relative luminescence by 39.14%. Altogether, these data suggested that CBPA could improve T-cell function by interfering with the PD-1/PD-L1 immune checkpoint pathway.

### 2.5. CBPA Activated the Function of CD4^+^ T Cells In Vitro

**Figure 5:**
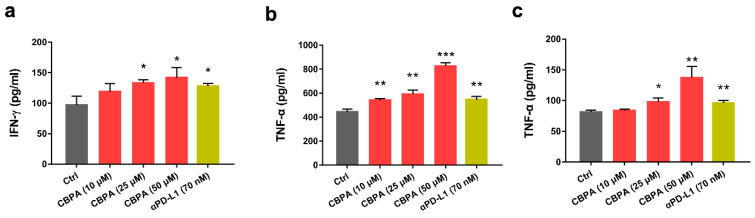
Effects of CBPA on T-cell activity and cytokine level. (**a**,**b**) CBPA rescued IFN-γ and TNF-α production in CD4^+^ T-cells co-cultured with hPD-L1-overexpressed HEK293T cells. (**c**) Effects of CBPA on TNF-α secretion from CD4^+^ T-cells co-cultured with NCI-H1975 cells. Data are shown as means ± SEM, * *p* < 0.05, ** *p* < 0.01, *** *p* < 0.001, unpaired *t*-test.

Cytokine production is an important indicator for T-cell activation evaluation. To further investigate whether CBPA can restore the repressed function of T cells, we evaluated the production of IFN-γ and TNF-α from CD4^+^ T cells using a target cell/T cell co-culture assay. Briefly, activated CD4^+^ T cells were co-cultured with well-established HEK293T cells stably expressing human PD-L1, which was followed by the addition of CBPA and the anti-PD-L1 antibody atezolizumab at the indicated concentrations. A dose-dependent increase in IFN-γ and TNF-α secretion was found with CBPA treatment (Figure 5a,b). In the presence of 50 μM CBPA, the IFN-γ level was increased by 46.49%, TNF-α production was increased by 86.11% compared to the vehicle-treated controls. In addition, the enhanced intracellular cytokine secretion with the addition of CBPA was verified by a NCI-H1975 cell/T-cell co-culture assay. Similarly, compound CBPA significantly enhanced TNF-α secretion in a dose-dependent manner. As can be evaluated from Figure 5c, the addition of CBPA at a 50 μM concentration increased the TNF-α level by 68.07%. Therefore, consistent with the results of the luciferase reporter assay, CBPA could rescue PD-L1-inhibited T-cell activity.

### 2.6. Antitumor Efficacy of the CBPA in Tumor-Bearing C57BL/6 Mice

**Figure 6:**
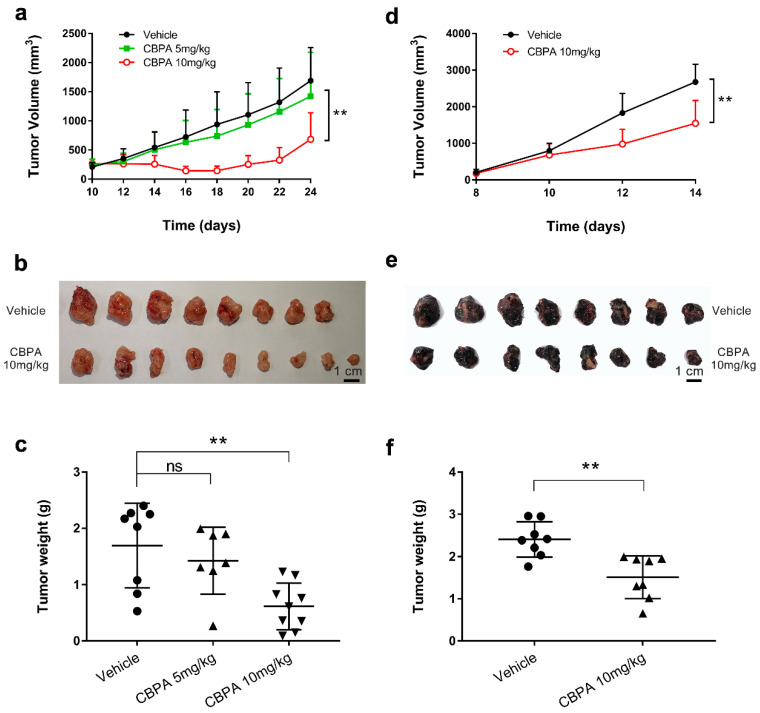
In vivo efficacy study of the CBPA in the tumor-bearing C57BL/6 mice models. (**a**) MC38 tumor volume changes during treatment with vehicle (n = 8), 5 mg/kg CBPA (n = 7), and 10 mg/kg CBPA (n = 9), respectively. (**b**,**c**) The images and weights of excised MC38 tumors in each group. (**d**) B16F10 tumor volume changes during treatment with vehicle (n = 8) and 10 mg/kg CBPA (n = 8), respectively. (**e**,**f**) The images and weights of excised B16F10 tumors in each group. All quantitative data are represented as means ± SEM, ** *p* < 0.01, unpaired *t*-test.

To verify whether CBPA can exert antitumor activity in vivo, we implanted mice with MC38 colon adenocarcinoma and B16F10 melanoma tumor models, respectively; allowed the tumors to become established; and then injected vehicle and CBPA via intraperitoneal injection every other day. The animals were then monitored for tumor growth at the injected site. CBPA at 5 mg/kg showed a slight effect on tumor growth inhibition (TGI) in MC38-bearing C57BL/6 mice (Figure 6a–c), while with administration of CBPA at a dose of 10 mg/kg, animals had significantly lower eventual total tumor burdens. The average tumor weight for the 10 mg/kg CBPA treated group was 0.62 ± 0.14 g, which was significantly lower than that of the vehicle control (1.70 ± 0.27 g). Furthermore, the TGI of the 10 mg/kg CBPA-treated group was 67.20% greater than the vehicle control. Notably, the CBPA compound was effective not only against colon adenocarcinoma but also against other histologic types, such as melanoma (B16F10) (Figure 6d–f). CBPA at 10 mg/kg exhibited significantly higher TGI compared to the vehicle control at 45.26% and showed an average tumor weight of 1.51 ± 0.18 g, significantly lower than that of the vehicle control (2.41 ± 0.15 g). During the treatment period, no obvious signs of toxicity or body weight loss were seen in the mice. Moreover, the serum biochemical parameters of liver and kidney function in mice were analyzed to further evaluate the safety properties of CBPA. The serum biochemical indices, including TP, ALB, GLO, AST, ALT, ALP, BUN, Cr, and UA, showed no significant changes upon CBPA treatment at doses up to 50 mg/kg, confirming that CBPA treatment caused no observable toxicity in murine models (Supplementary Figure S5). 

### 2.7. CBPA Alters Gene Signatures in Murine Tumor Tissue

**Figure 7:**
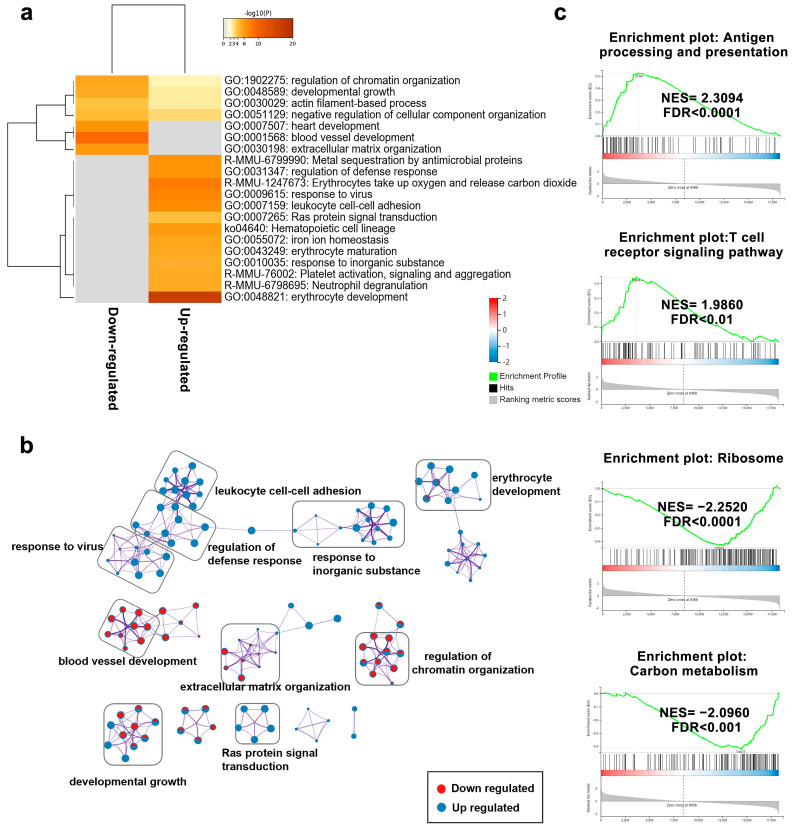
Effects of CBPA on gene expression signatures in tumor tissue. (**a**) Heatmap from Metascape analysis displaying the top enrichment pathways of down- and upregulated transcripts in MC38 tumor tissue excised from CBPA-treated (10 mg/kg, n = 3) group. Gray color indicates no significance. (**b**) Functional network analyses based on differentially expressed transcripts were performed using the Metascape database. Red/blue nodes indicate the terms with down-/upregulated transcripts, respectively. The sizes of the nodes show the significance of the enrichment results. (**c**) Plotting of the enriched KEGG pathways using GSEA.

Unbiased transcriptome profiling was performed using RNA sequencing to assess the differences in the transcriptional signatures between MC38 tumor tissue isolated from CBPA- (10 mg/kg) and vehicle-treated mice (Figure 7). Compared to the vehicle-treated group, 942 differentially expressed transcripts were identified in the CBPA-treated group, including 373 downregulated transcripts and 569 upregulated transcripts (Supplementary Table S2). To investigate the biological processes and pathways that may be affected by CBPA treatment, the Metascape database was applied for functional enrichment analysis. The results indicated that the upregulated transcripts in the CBPA-treated group were mainly involved in the regulation of defense response, leukocyte cell–cell adhesion, and erythrocyte development. In comparison, biological pathways, such as developmental growth, blood vessel development, and extracellular matrix organization, were found to be downregulated in the CBPA-treated group (Figure 7a,b). In view of the fact that the gene expression differences between the CBPA treatment group and the vehicle control group were minimal, GSEA [32] was subsequently performed. The GSEA also revealed that several immune pathways, such as antigen processing and presentation, natural killer cell-mediated cytotoxicity, Th1 and Th2 cell differentiation, cytokine–cytokine receptor interaction, T-cell receptor signaling, and Th17 cell differentiation, were significantly upregulated, while pathways related to ribosome and carbon metabolism were significantly downregulated, in CBPA-treated mice (Figure 7c and ). Overall, these results demonstrated that CBPA treatment played an important role in antitumor immune response.

### 2.8. Antitumor Efficacy of the CBPA via a T-Cell-Dependent Mechanism

**Figure 8:**
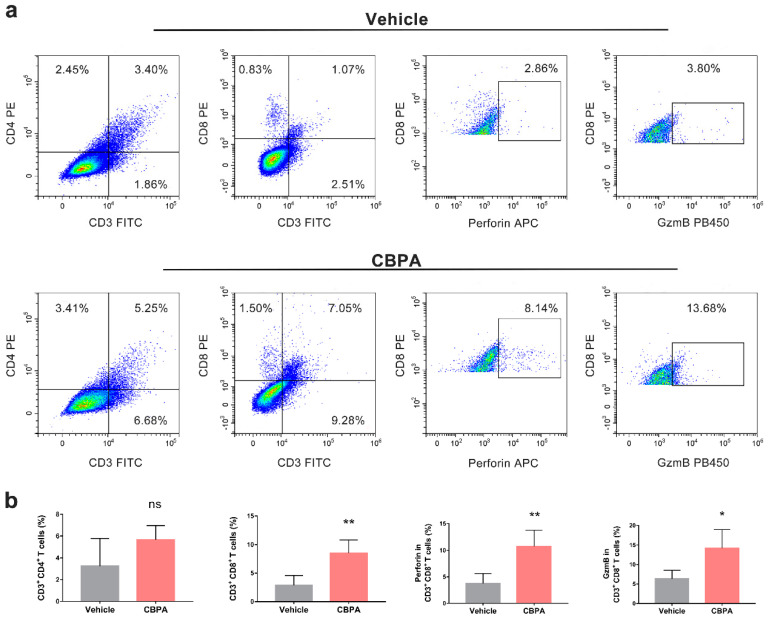
The in vivo antitumor mechanism of CBPA. (**a**) Flow cytometry analysis of TILs and intracellular perforin and GzmB secretion in tumor tissues collected from MC38-bearing mice administered CBPA (10 mg/kg) or the vehicle. (**b**) All quantitative data from the flow cytometry analysis (n = 4) are shown as means ± SEM, * *p* < 0.05; ** *p* < 0.01, unpaired *t*-test; ns, not significant.

**Figure 9:**
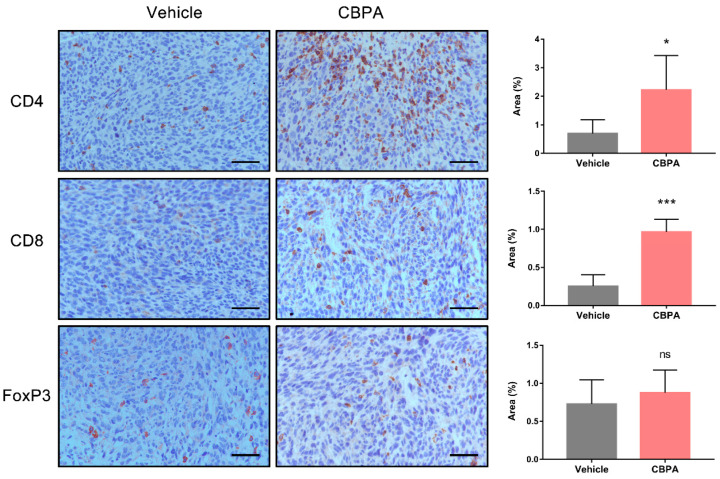
Immunohistochemical analysis of CD4, CD8, and FoxP3 expression in tumor tissues isolated from CBPA- (10 mg/kg) and vehicle-treated MC38 tumor-bearing mice. Scale bars = 100 μm, ×200. Immunohistochemical quantification (n = 4) was undertaken using Image J. Data are shown as means ± SEM, * *p* < 0.05; *** *p* < 0.001; ns, not significant, unpaired *t*-test.

Next, we evaluated CBPA-mediated antitumor responders, including tumor-infiltrating lymphocytes (TILs) and secretion of proinflammatory cytokines in the TME. Tumors tissues were collected from MC38-bearing mice treated with CBPA or the vehicle and TILs were examined with FACS and IHC analysis. As illustrated in Figure 8a,b, the infiltration of CD3^+^CD4^+^ T cells modestly increased and the percentages of CD3^+^ CD8^+^ T cells significantly increased in the CBPA-treated tumors. Similarly, the IHC analysis confirmed a significant increase (*p* < 0.05) in CD4^+^ and CD8^+^ T-cell population infiltration in the TME in response to CBPA at a dose of 10 mg/kg (Figure 9). In contrast we did not observe a significant change for the expression level of FoxP3, which is a valid biomarker for Treg cells. We also assessed the CBPA-dependent induction of perforin and GzmB production by intratumoral CD8^+^ T cells. Using an intracellular staining approach with TILs collected from CBPA-treated groups (10 mg/kg), we demonstrated a significant increase in perforin and GzmB production (Figure 8). Altogether, our data confirmed that CBPA mainly elicited an antitumor immune response via increased TILs and secretion of proinflammatory cytokines in the TME.

### 2.9. Binding Mode Prediction

**Figure 10:**
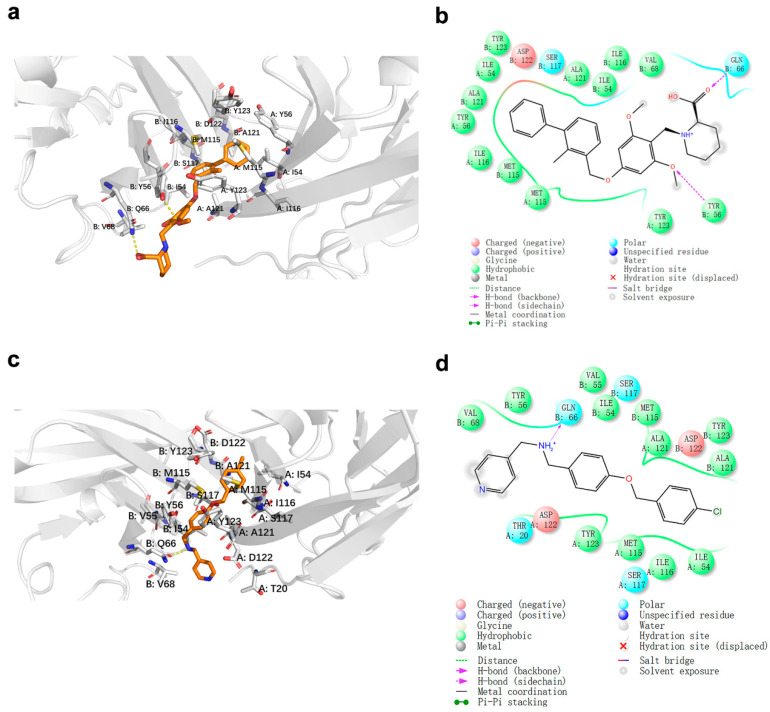
Binding mode of inhibitor 1 and CBPA. Two-dimensional (2D) model diagram of PD-L1 with (**a**) inhibitor 1 and (**c**) the CBPA compound and 3D binding mode of PD-L1 with (**b**) inhibitor 1 and (**d**) CBPA.

To demonstrate the mode of binding of CBPA to hPD-L1, the docking poses generated by Glide SP were analyzed. The structure of dimeric PD-L1 (PDB ID: 5J89) bound to inhibitor 1 demonstrated that inhibitor 1 formed hydrophobic interactions with amino-acid residues I54, Y56, M115, I116, A121, and Y123 and key hydrogen bonds with Q66 and Y56 of the B chain (Figure 10a,b). As shown in Figure 10c,d, CBPA, which could be sandwiched into the center of the hydrophobic cleft formed by dimeric PD-L1, formed hydrophobic interactions with residues I54, V55, Y56, V68, M115, I116, A121, and Y123. CBPA also formed a key hydrogen bond with residue Q66 of the B chain, which stabilized the complex. Moreover, the docking scores of inhibitor 1 and CBPA were −9.591 kcal/mol and −8.944 kcal/mol, respectively, which was consistent with the previous experimental results. The results demonstrated that CBPA shows a similar binding mode as BMS compounds. 

### 2.10. Binding Free Energy Calculation

**Figure 11:**
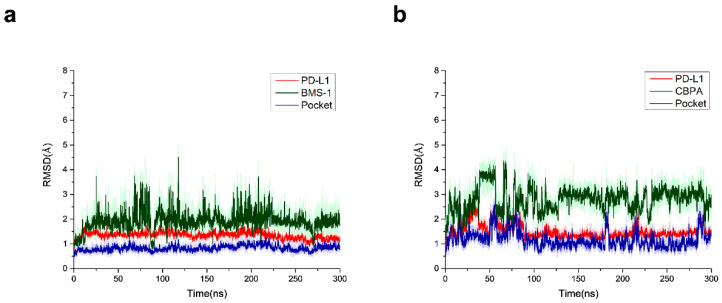
RMSDs of the pocket backbone and the ligands’ heavy atoms in the two systems during MD simulations. (**a**) RMSD of the backbone atoms of the PD-L1 pocket and the heavy atoms of inhibitor 1 (BMS-1). (**b**) RMSD of the backbone atoms of the PD-L1 pocket and the heavy atoms of CBPA.

Molecular dynamics (MD) simulations (300 ns) were performed for the two ligand-protein complexes, and the stability of each system was assessed by monitoring the RMSDs of the backbone atoms of the PD-L1 pocket and the heavy atoms of the ligands. The RMSD plots showed that both of the two systems reached relative stability after ~250 ns, indicating that the two systems had been equilibrated (Figure 11).

Subsequently, the total binding free energies (ΔG_bind_) of CBPA and inhibitor 1 with hPD-L1 were calculated by using the MM/GBSA method (Table 1). The ΔG_bind_ values for inhibitor 1 and CBPA were −29.37kcal/mol and −24.63 kcal/mol, respectively, which was in agreement with the order of the experimental values. The results also indicated that van der Waals interaction and electrostatic interaction were the main contributors to the interactions between PD-L1 and inhibitors, while the polar interaction was unfavorable for the binding. The main difference affecting the ΔG_bind_ value between the CBPA and the inhibitor was the electrostatic interaction (−107.70 kcal/mol for CBPA and −16.28 kcal/mol for inhibitor 1).

The ΔG_bind_ of the dimeric hPD-L1 bond to the inhibitor was decomposed into that of each amino-acid residue to investigate the detailed contributions of key residues in the complex structure.

A single residue with an energy contribution value of less than −1 kcal/mol was considered to be critical. In the inhibitor 1/PD-L1 system, residues Y56, M115, A121, and Y123 of chain A and I54, Y56, Q66, M115, and A121 of chain B provided strong contributions to the ligand binding (Supplementary Figure S6a). In the CBPA/PD-L1 system, residues M115 and D122 of chain A and I54, Y56, M115, and A121 of chain B played critical roles in the ligand binding (Supplementary Figure S6b). Thus, residues I54, Y56, Q66, M115, A121, D122, and Y123, were identified as the key residues for the ligand binding to PD-L1, and it was hoped that they would offer some clues for the virtual screening and design of novel PD-1/PD-L1 inhibitors.

## 3. Discussion

The design and synthesis of small-molecule inhibitors of PD-1/PD-L1 is a considerably desirable strategy that would add to existing cancer immunotherapies. However, due to the fact that the PD-1/PD-L1 interface is a relatively large, flat, and highly hydrophobic interface without well-defined pockets, the discovery of small-molecule inhibitors that could interrupt the interaction of PD-1/PD-L1 is inherently challenging [22,33]. Indeed, potential compounds with new skeletons are still needed to design novel anticancer agents that can generate a durable antitumour immune response in patients who do not benefit from the existing drugs. The docking-based virtual screening approach is a key strategy for identifying candidate compounds with potential activity from small-molecule databases [34]. We present here the in silico identification and experimental validation of small-molecular agents with activity against PD-1/PD-L1 PPI based on the crystal structure of the ligand bond to PD-L1 (PDB code: 5J89). After screening a series of small-molecule compounds, we determined that the compound CBPA can block the PD-1/PD-L1 interaction, eliciting an antitumor response by acting to reinvigorate T-cell cytotoxicity.

A major challenge in tumor immunotherapy is breaking tumor immune tolerance [4]. In the present study, the bioactivity of CBPA was comprehensively evaluated with in vitro and in vivo platforms. We used SPR as a detection method and the K_D_ value as the parameter for the affinity between CBPA and hPD-L1. In this study, CBPA showed determinable hPD-L1 binding affinities, presenting K_D_ values at the micromolar level (Figure 2). Furthermore, CBPA also showed ligand-blocking activation and induced significant, dose-dependent increases in cytokine release in cell-based assays. Utilizing well-established murine colon adenocarcinoma and melanoma tumor models, we demonstrated that CBPA at a dose of 10 mg/kg significantly inhibited the growth of subcutaneous tumors in terms of both tumor weight and volume, and its inhibitory rate in terms of tumor volume was approximately 50% (Figure 6). These data demonstrated that, in addition to in vitro efficiency, CBPA has antitumor potency as a single agent in multiple tumor models.

Tumor-infiltrating lymphocytes are key modulators in antitumor response, and their function is significantly affected by the tumor microenvironment [35]. The blockage of inhibitory receptors can restore and even enhance the function of TILs [36], which leads to the priming of a large number of antigen-specific activated CD8^+^ cytotoxic T cells and CD4^+^ helper T (TH) cells for effector functions in the elimination of malignant cells. The activated CD8^+^ T cells in the tumor microenvironment can directly kill tumor cells through secretion of cytotoxic cytokines, such as granzyme and perforin, which leads to apoptosis of the tumor cells [37,38,39]. In our study, as shown by the RNA-seq data, CBPA induced enhanced antitumor immune responses characterized by increased antigen processing and presentation, natural killer cell-mediated cytotoxicity, Th1 and Th2 cell differentiation, cytokine–cytokine receptor interaction, T-cell receptor signaling, and Th17 cell differentiation (Figure 7 and Supplementary Table S3). Consistent with the RNA-seq results was the finding that, in the MC38 tumor-bearing mice, CBPA treatment could increase CD4^+^ and CD8^+^ T-cell infiltration in the tumor tissue, which may have been caused by T-cell proliferation (Figure 8 and Figure 9). Here, we used the expression levels of GzmB and perforin to indicate the degree of activation of CD8^+^ cytotoxic T cells. The results showed that CBPA treatment could produce a significant increase in perforin and GzmB production by intratumoral CD8^+^ T cells. Thus, due to the above data, we believe that CBPA can interrupt PD-1/PD-L1 PPI, eventually suppressing tumor growth by acting on the tumor microenvironment. We also validated the unspecific toxicity of CBPA using cell viability assays in which MC38 and B16F10 cell lines were incubated with a concentration gradient of CBPA for 48 h. As shown in Supplementary Figure S7, CBPA could reduce the viability of MC38 and B16F10 cells in a dose-dependent manner, with IC_50_ values of 57.67 and 77.45 μM, respectively, at 48 h. Thus, we speculated that CBPA may have other targets in addition to PD-L1 collaboratively implicated in the antitumor response. Based on SPR experiment, we found no binding of CBPA to hPD-1. Our future work will try to optimize the structure of CBPA for increasing PD-1/PD-L1 blocking efficiency.

Autoimmune toxicities appear to occur more frequently with systemically administered immune checkpoint antibodies [40,41,42]. Numerous clinical trials have found that enhanced efficacy for antibodies is often accompanied by increased side effects in patients, so developing novel, potent compound inhibitors and combinatory therapies represents an appealing avenue for cancer therapy [43]. Moreover, in order to develop a suitable candidate agent with a good balance of high activity and negligible toxicity, the pharmacodynamic and pharmacokinetic properties of the compound inhibitors should be studied in-depth. Notably, we found that the therapy was well-tolerated when the animals received 50 mg/kg CBPA daily (Supplementary Figure S5). We did not observe remarkable drug-related deaths or adverse effects during the toxicity experiment. Therefore, these data suggested that CBPA could act as a hit compound for the further development of potent PD-1/PD-L1 compound inhibitors, and further in-depth mechanistic studies of this compound are required. 

In conclusion, using a docking-based virtual screening followed by a series of experimental verifications, we demonstrated that the compound CBPA can bind to hPD-L1, effectively inhibit the PD-1/PD-L1 axis, and thereby reinvigorate PD-L1-mediated T-cell-response repression. Moreover, CBPA displayed significant in vivo antitumor efficacy and negligible toxicity, making it a new potential hit compound worthy of further investigation.

## 4. Materials and Methods

### 4.1. Preparation of Crystal Structures and Datasets

The crystal structures of the dimeric PD-L1 protein complexed with the inhibitor were downloaded from the PDB database, including 5J8O, 5N2D, 5N2F, and 5J89. For each crystal structure, the Protein Preparation Wizard panel in the program developed by Schrödinger in 2015 (Schrödinger, LLC, New York, NY, USA, 2015) was applied to add hydrogen and missing side chains, eliminate all water molecules, assign partial charges and protonation states, and minimize the structure with the OPLS2005 force field. The crystalline water molecules and ions that were distant from the ligand and exceeded 3 Å in each crystal structure were removed. The sampling power was evaluated using cross-docking calculations [44]. Molecular docking was performed using the Glide program with both standard and extra precision. The RMSD between the heavy atoms of the crystal structure pose and docking pose was calculated and used as the standard to evaluate the conformation sampling ability. To evaluate the virtual screening capability of these four crystal structures, seven reported active inhibitors [45] (BMS-8, 37, 200, 202, 242, 1001, 1166) served as a validation dataset, and their decoys, generated with an active-to-decoy ratio of 1:50 with DUD•E [46], were considered as a decoy dataset.

### 4.2. Virtual Screening and Molecular Docking

The Specs database consisting of more than 200,000 compounds was used as small-molecule database for virtual screening. Firstly, after the initial compound-screening process, which included desalinization, neutralization processing, and PAINS filtration through the comprehensive application of the Molinspration Cheminformatics software Open Eye, Chem Axon, RDkit, and CERTARA, a total of 120,000 compounds were selected for further analyses. The ligand-based 3D pharmacophore models were generated using the Phase module in the Schrodinger software package based on the co-crystallized structures of hPD-L1with the reported inhibitors BMS-8, BMS-37, BMS-200, and BMS-202 (PDB: 5J8O, 5N2D, 5N2F, 5J89), respectively. The key chemical features of having one positive center, one hydrogen bond acceptor, one hydrophobic center, and three aromatic ring centers were chosen to construct an effective pharmacophore model for virtual screening. Following this, 10,125 compounds satisfied the condition of having at least four pharmacophore features among the 120,000 compounds.

The ROC curves and AUC values were employed to evaluate the virtual screening abilities of the four co-crystallized structure and, as a result of this, 5J89 was finally selected as the initial structure for the final virtual screening. The generated grid and prepared ligands were then subjected to docking-based virtual screening using Glide. Through two-level (SP and XP) screenings, 1110 top-ranked compounds with docking XP Gscores ≤ −9.000 kcal/mol were obtained. Furthermore, we used the clustering protocol in the Canvas module to cluster these molecules into 402 groups with Tanimoto coefficients of less than 0.5. The compounds with the best scores in each group were retained and subjected to fully flexible docking using the Induced Fit module. Finally, a total of 91 chemicals were selected and purchased from Topscience Co., Ltd. (Shanghai, China) for subsequent bioactivity validation.

The detailed interactions between the small molecule CBPA and PD-L1 were subjected to further docking calculations. The crystal structure of PD-L1 complexed with a ligand (PDB ID: 5J89) was retrieved as a docking model, and again prepared using the Protein Preparation Wizard in Schrödinger. The structures of the CBPA, inhibitor 1 and co-crystal ligand were respectively constructed and minimization was applied using Chemdraw and Chem3D software. Then, the ligand was prepared in LigPrep with an OPLS-2005 force field to generate the corresponding low-energy 3D conformers. The docking grid box was generated to define the active binding site. The CBPA, inhibitor 1 and co-crystal ligand were docked without bias into the PD-L1 binding site using the Glide module with SP. The binding mode was analyzed based on the pose with the best score. 

### 4.3. Surface Plasmon Resonance Assay

SPR experiments were performed with a Biacore X100 (GE Healthcare) equipped with a CM5 sensor chip (Series S Sensor Chip, GE Healthcare) at 25 °C. The PD-L1 protein was covalently immobilized on the sensor surface at ~6000 response units (RUs) by using standard amine-coupling in 10 mM sodium acetate (pH 5.5). For the binding experiments, 1.05 × PBS buffer (pH 7.4, GE Healthcare) supplied with 0.05% surfactant P20 and 5% DMSO (Sigma-Aldrich) was used as the running buffer. CBPA purchased from TopScience Co., Ltd. (Shanghai, China) was dissolved in DMSO. A calibration procedure was carried out to eliminate variations in the bulk responses between samples caused by the presence of high-refractive-index DMSO [47,48]. The CBPA was serially diluted with PBS buffer and given a circle for 60 s in the contact phase followed by 60 s in the dissociation phase, using a flow rate of 30 μL/min throughout the experimentation. The changes in the response level detected during the analysis were corrected based on the DMSO calibration plot. The sensor grams were globally analyzed with a steady-state model using Biacore T200 Evaluation software, version 2.0 (GE Healthcare).

### 4.4. Cell Culture

Jurkat, NCI-H1975, B16F10, and HEK293T cells were obtained from ATCC; the MC38 cell line was obtained from Crisprbio Biotechnology company (Beijing, China); and the primary CD4^+^ T cells with purity above 95% were obtained from LDEBIO company (Guangzhou, China). NCI-H1975 and Jurkat cells were maintained in RPMI-1640 medium supplemented with 10% fetal bovine serum (FBS) and 1% penicillin/streptomycin. MC38, B16F10, and HEK293T cells were maintained in DMEM medium supplemented with 10% FBS and 1% penicillin/streptomycin. All cells were incubated in a humidified atmosphere with 5% CO_2_ at 37 °C. To generate CD4^+^ T-cell blasts, cells were maintained in RPMI-1640 medium with 15% FBS and IL-2 (100 IU/mL; 202-IL-010, R&D). T-Activator CD3/CD28 Dynabeads for T Cell Expansion and Activation (11131D, Gibco) were added into the culture medium at a bead-to-cell ratio of 1:1 and incubation was implemented in a humidified CO_2_ incubator at 37 °C. After approximately 4–7 days, the activated CD4^+^ T cells were harvested for further experiments. 

### 4.5. Flow Cytometry Analysis

Jurkat cells stably over-expressing hPD-1 (Jurkat-hPD-1) were engineered and used to examine the PD-1/PD-L1 blocking activity of CBPA with flow cytometry at the cell level. Different pre-incubated solutions was added into five tubes (tube 1: 100 µL PBS as the negative control; tube 2: 50 µL commercially available recombinant hPD-L1-His protein (10 ug/mL, 10084-H08H, Sino Biological) + 50 µL PBS as the positive control; tube 3: 50 µL PD-L1-His protein + 50 µL 40 µM inhibitor 1 compound (BMS-1 compound in WO 2015/034820A1 patent [19], Selleck, S7911); tubes 4 to 5: 50 µL PD-L1-His protein + 50 µL 200 µM or 40 µM CBPA compound) and incubated for 30 min at 4 °C. Meanwhile, cells were harvested, washed with PBS, and re-suspended in pre-cooling PBS at a concentration of 4 × 10^6^ cells/mL. Then, 100 µL of cells was added to each of the above tubes, and they were mixed gently and co-incubated on ice for an additional 60 min. Subsequently, cells were harvested, washed twice with PBS, and stained with Anti-His-APC (BioLegend) in buffer for 20 min in the dark on ice. Stained cells were washed once and measured on a flow cytometer (CytoFLEX S, Beckman), and the data were analyzed by using FlowJo software (TreeStar, Ashland, OR, USA). We calculated the blocking efficiency (%) with the formula: 100% × (1 − positive rate _treatment_/positive rate _positive control_).

### 4.6. Luciferase Based PD-1/PD-L1 Blockade Assay

The luciferase reporter assay was assessed as described [49]. For the assay, two genetically modified cell lines preserved in our laboratory were used, including a surrogate of antigen-presenting cells (HEK293T cell line overexpressing PD-L1 and the TCR agonist; referred to as hPD-L1 aAPCs) and PD-1 effector cells (Jurkat T cells overexpressing PD-1 and containing a luciferase reporter gene under the control of the TCR-inducible NFAT promoter; referred to PD-1 effector cells). Briefly, 10,000 hPD-L1 aAPC cells were seeded into 96-well plates and allowed to adhere overnight. The following day, after removing the medium from the wells, serial dilutions of the tested compound and control antibody nivolumab (Selleck, China) were prepared in RPMI-1640 with 10% FBS and added to the wells containing hPD-L1 aAPC cells. Immediately, 20,000 PD-1 effector cells were separately added per well in the same medium. The co-culture was then incubated for 6 h at 37 °C and equilibrated for 30 min at room temperature. Bio-Glo reagent (Promega) was added to the wells in a 1:1 ratio and the luminescence was measured on a microplate reader (Enpire, PE) after a further 10 min incubation.

### 4.7. ELISA for Intracellular Cytokine Detection

T-cell response assays were conducted by ELISA based on the target cell/T-cell co-culture assay. Human CD4^+^ T lymphocytes were expanded and activated as described above. NCI-H1975 cells or HEK293T cells with hPD-L1-stable overexpression (HEK293T-hPD-L1) were seeded in 96-well plates with a density of 1 × 10^4^ cells/well and allowed to adhere for 24 h. Subsequently, NCI-H1975 or HEK293T cells were co-cultured with activated CD4^+^ T lymphocytes at a ratio of 1:2, which was followed by the addition of the additives (Atezolizumab or CBPA) to acquire a total complete RPMI-1640 medium of 200 μL and co-incubation at 37 °C in a 5% CO_2_ incubator for 48 h. Culture supernatants were harvested for the detection of IFN-γ and TNF-α levels by using a human IFN-γ ELISA kit (Cat. 88-7316-22, Invitrogen) and human TNF-α ELISA kit (Cat. 88-7346-88, Invitrogen, Carlsbad, CA, USA), respectively.

### 4.8. Cell Viability Assays

A Cell Counting Kit 8 (Beyotime, Shanghai, China) was used for the cell cytotoxicity assay. Briefly, 5 × 10^3^ cells were plated into transparent 96-well plates with 100 μL of media per well and cultured for 48 h in the presence of increasing concentrations of the CBPA compound. Then, 10 μL of Cell Counting Kit 8 reagent was added to each well and the cells were incubated for another 2 h at 37 °C. Absorbance at 450 nm was measured with a microplate reader (Enpire, PE).

### 4.9. Western Blot Analysis

Cells were harvested and total protein was extracted using RIPA buffer containing 50 mM Tris-HCl (pH 7.4), 150 mM NaCl, 1% Nonidet P-40, 0.25% sodium deoxycholate, and 1 mM EDTA supplemented with a protease and phosphatase inhibitor cocktail (Bimake, Shanghai, China). Western blotting was performed as previously described [50]. Primary antibodies against hPD-1, hPD-L1, and GAPDH were purchased from Abcam Inc. (Cambridge, MA, USA).

### 4.10. In Vivo Effect of CBPA

Six- to eight-week-old female C57BL/6 mice were purchased from Weitong Lihua Experimental Animal Technical Co., Ltd. (Beijing, China). All animals were housed under pathogen-free conditions in the Laboratory Animal Facility of School of Basic Medical Sciences, Henan University. All animal studies were approved by the Medical and Scientific Research Ethics Committee of Henan University and conducted in strict accordance with the guidelines for the Care and Use of Laboratory Animals of the Ministry of Science and Technology of the People′s Republic of China (2006-398).

MC38 and B16F10 tumor cells (3 × 10^6^ cells in PBS) were subcutaneously implanted into the right flank of the mice. When the mean tumor volume reached approximately 50–70 mm^3^, mice were randomly divided into the experimental groups. Then, mice were dosed intraperitoneally with the vehicle (normal saline containing 5% DMSO) or CBPA compound every other day. Tumor growth was monitored with a digital caliper every two days, and the tumor volumes were calculated according to the formula: tumor volume (mm^3^) = 1/2 × length × width^2^. Mice were sacrificed when body weight had >20% loss, and then the tumor tissue, blood, and major organ (liver and spleen) samples were collected for immune phenotype and function analyses.

### 4.11. RNA-Seq and Data Analysis

At the end of the experiment, MC38 tumors from mice treated with CBPA (10 mg/kg) or the vehicle control were harvested. Total RNA samples were isolated from frozen tissues using TRIzol Reagent (Invitrogen, USA) and quantified with NanoDrop (Thermo Fisher Scientific, USA). Sequencing libraries were prepared using an MGI Easy™ mRNA Library Prep Kit (BGI, Wuhan, China) according to the manufacturer’s instructions. RNA-seq analysis was performed on the BGIseq500 platform (BGI-Shenzhen, China). For the data processing, quality trimming of the reads was performed using SOAPnuke (v1.5.2) [51] to remove sequencing-adapter and low-quality bases. Clean reads were aligned to the Mus musculus genome sequence (Mus musculus. GRCm38) using HISAT2 (v2.0.4) [52]. Subsequently, Bowtie2 (v2.2.5) [53] was used to align the clean reads to the reference coding gene set, and then RSEM (v1.2.12) [54] was applied to calculate the expression levels of genes. Furthermore, differentially expressed gene (DEG) analysis were performed using DESeq2 (v1.4.5) [55] with Q values ≤ 0.05. 

In order to explore the change in phenotype, comprehensive functional enrichment analysis of DEGs was annotated using Metascape [56]. Gene set enrichment analysis (GSEA) [32] was used to extract the specific gene expression patterns and obtain biological insights. The significant levels of the terms and pathways were determined by Q value with a rigorous threshold (Q value ≤ 0.05).

### 4.12. Analysis of Tumor-Infiltrating Lymphocytes

At the end of the experiment, excised MC38 tumors from mice treated with CBPA (10 mg/kg) or the vehicle control were digested into single-cell suspensions. Cells were washed, resuspended in FACS buffer, and used as antibodies against surface antigen staining for 30 min at 4 °C with FITC anti-mouse CD3 (Cat. 100203, BioLegend, San Diego, CA), PE anti-mouse CD4 (Cat. 100407, BioLegend, San Diego, CA), and PE anti-mouse CD8 (Cat. 100707, BioLegend, San Diego, CA). Cells were then fixed and permeabilized, stained for perforin (Cat. 154303, BioLegend, San Diego, CA) and granzyme B (Cat. 396413, BioLegend, San Diego, CA), and analyzed by flow cytometry.

### 4.13. Immunohistochemistry (IHC) Staining

At the end of the experiment, excised MC38 tumor tissues were fixed in 4% paraformaldehyde, embedded in paraffin, and sectioned with 4 µm thickness. After deparaffinization and rehydration, the sections were incubated overnight at 4 °C with primary antibodies for CD8 (Cat. ab209775, Abcam), CD4 (Cat. ab183685, Abcam), or FoxP3 (Cat. ab215206, Abcam). The tissue sections were incubated with the HRP-labeled secondary antibody for 30 min and counterstained with DAB. Finally, images from random fields were obtained using a Nikon fluorescence microscope (NI-U, NIKON). The expression levels of CD8, CD4, and FoxP3 were quantified by counting the numbers of positive particles under magnification.

### 4.14. Biochemical Analysis

For the toxicity study, healthy C57BL/6 mice were randomized and dosed daily with the vehicle control or 50 mg/kg of the CBPA, respectively. On day 8 post-first dose, serum samples from the animals were analyzed for the levels of total protein (TP), albumin (ALB), globulin (GLO), aspartate aminotransferase (AST), alanine transaminase (ALT), alkaline phosphatase (ALP), blood urea nitrogen (BUN), creatinine (Cr), and uric acid (UA) using an automatic dry-type biochemical analyzer (DRI-CHEM NX500iVC, FUJI). 

### 4.15. Statistical Analysis

Data were presented as means ± standard deviation from independent triplicate experiments. Prism 7.0 software (GraphPad) was used to statistically analyze the significance of differences between groups via an unpaired Student’s *t* test. *p* values < 0.05 were considered statistically significant.

### 4.16. Molecular Dynamics Simulation

To demonstrate the binding mechanism of the compound bond to PD-L1, MD simulations were performed for the hPD-L1 in complex with inhibitor 1 and CBPA. The initial structure of the dimeric PD-L1-CBPA for the MD simulation was obtained from Glide docking. The restrained electrostatic potential (RESP) calculated with the Hartree–Fock (HF) method with a 6-31G* basis set in the Gaussian software was used to fit the partial charges for the inhibitors. The ff99SB and the gaff force field were used to parameterize the protein and the inhibitors, respectively. The protein–ligand system was solvated in TIP3PBOX at a distance of 12 Å to the boundary. After adding sodium ions to neutralize the systems, the systems were minimized, heated, and equilibrated. MD simulations were performed at 300 K with 1.0 atmospheric pressure in an NPT ensemble. In the simulation process, the PME method [57] was used to calculate the long-range electrostatic interaction, and the SHAKE algorithm [58] was used to constrain the bond length containing the hydrogen bond. 

### 4.17. Binding Free Energy Calculation

The binding free energy (ΔG_bind_) between the receptor and ligand in each system was calculated with the molecular mechanics generalized Born surface area (MM/GBSA) method [59,60,61]. A total of 1000 snapshots extracted from the last 50 ns trajectories of the system were employed to analyze the binding free energy using AMBER14. A MM/GBSA decomposition analysis was performed to decompose the total free energy into the contributions of individual inhibitor–residue pairs to identify the key residues. To reduce the computational demand, 50 of the 1000 snapshots were selected to calculate the entropy term (−TΔS).

## Figures and Tables

**Table 1:** ΔG_bind_ for the two inhibitors (inhibitor 1 and CBPA) bound to PD-L1 with the MM/GBSA method (kcal/mol).

Terms	PD-L1/Inhibitor 1	PD-L1/CBPA
Δ*G_ele_*^a^	−16.28	−107.70
Δ*G_vdW_*^b^	−62.63	−50.02
Δ*G_nonpol,sol_*^c^	−6.77	−5.84
Δ*G_pol,sol_*^d^	28.81	120.67
Δ*H* ^e^	−50.10	−37.06
−*T*Δ*S* ^f^	20.73	12.43
ΔG*_cal_*^g^	−29.37	−24.63
ΔG*_exp_*^h^	−9.49	−5.97

^a^ Electrostatic interaction contribution; ^b^ van der Waals contribution; ^c^ non-polar solvation free energy; ^d^ polar solvation free energy; ^e^ enthalpy change; ^f^ entropy change contribution; ^g^ calculated Gibbs free energy; ^h^ experimentally measured Gibbs free energy.

## Data Availability

The data presented in this study are available in the article.

## Supplementary Materials

The following supporting information can be downloaded at: https://www.mdpi.com/article/10.3390/ijms24043971/s1.

## Abbreviations

TME	tumor microenvironment
SP	standard extra precision
RUs	response units
FBS	fetal bovine serum
DEGs	differentially expressed genes
GSEA	gene set enrichment analysis
TP	total protein
ALB	albumin
GLO	globulin
AST	aspartate aminotransferase
ALT	alanine transaminase
ALP	alkaline phosphatase
BUN	blood urea nitrogen
Cr	creatinine
UA	uric acid
PPI	protein–protein interaction
TILs	tumor-infiltrating lymphocytes
ΔG_bind_	total binding free energy
MD	molecular dynamics
RMSD	root-mean-square deviation
MM/GBSA	molecular mechanics generalized Born surface area
