# Supplementary material for: Identification of CBPA as a New Inhibitor of PD-1/PD-L1 Interaction
Source: Int J Mol Sci. 2023 Feb 16;24(4):3971. doi: 10.3390/ijms24043971 (PMC9964281; doi:10.3390/ijms24043971)
Supplement: Supplementary file 1 [file ijms-24-03971-s001.zip › supplementary/Supplementary Table S1.pdf]

Table S1. Results of cross-docking by the Glide SP mode and Glide XP mode

Results of cross-docking by the Glide SP mode

	RMSD			
Structure	5J8O	5J89	5N2D	5N2F
5J8O	0.80	14.87	15.65	18.71
5J89	16.42	1.39	1.21	7.83
5N2D	16.65	1.79	1.08	6.25
5N2F	16.23	3.29	2.94	4.19

Results of cross-docking by the Glide XP mode

	RMSD			
Structure	5J8O	5J89	5N2D	5N2F
5J8O	0.78	15.26	16.59	16.59
5J89	16.41	1.47	7.50	7.50
5N2D	16.52	1.05	3.54	3.54
5N2F	15.62	2.33	4.14	4.14
