# Supplementary material for: Identification of CBPA as a New Inhibitor of PD-1/PD-L1 Interaction
Source: Int J Mol Sci. 2023 Feb 16;24(4):3971. doi: 10.3390/ijms24043971 (PMC9964281; doi:10.3390/ijms24043971)
Supplement: Supplementary file 1 [file ijms-24-03971-s001.zip › supplementary/Supplementary Table S2.pdf]

Table S2. Transcripts differentially expressed in CBPA treated group compared to the vehicle control group

Gene Symbol	CBPA treated_ Expression	Control Expression	log2 (CBPA treated/Control)	Qvalue	Pvalue
Zfp395	0.0033	10.8033	-12.3191	0.0000	0.0000
Pigt	0.0000	10.2267	-11.3643	0.0000	0.0000
Son	0.0000	2.5600	-11.1550	0.0000	0.0000
Tln2	0.0000	1.3667	-10.8610	0.0000	0.0000
Gpr161	0.0000	1.9367	-10.4969	0.0000	0.0000
Fbf1	0.0033	2.5467	-10.3910	0.0000	0.0000
Tlk1	0.0000	3.3100	-10.3590	0.0000	0.0000
Gdnf	0.0000	2.4033	-10.2258	0.0000	0.0000
Ctif	0.0000	1.6600	-10.1316	0.0000	0.0000
Tmem106c	0.0000	6.2867	-9.9305	0.0000	0.0000
Ldlr	0.0000	1.9700	-9.9281	0.0000	0.0000
Mast3	0.0000	1.6100	-9.8884	0.0000	0.0000
Zfp516	0.0000	1.1067	-9.8578	0.0000	0.0000
Lrch3	0.0000	0.9433	-9.8442	0.0000	0.0000
Kif7	0.0067	1.8633	-9.8384	0.0000	0.0000
Ciz1	0.0000	3.3033	-9.8338	0.0026	0.0001
Mgat4a	0.0000	1.1167	-9.7807	0.0000	0.0000
Afap1l2	0.0000	1.9400	-9.7483	0.0000	0.0000
Zfp869	0.0000	2.3867	-9.6826	0.0000	0.0000
Cacna2d1	0.0000	1.0233	-9.6620	0.0000	0.0000
Utp4	0.0000	3.2900	-9.6502	0.0000	0.0000
Heg1	0.0000	0.7900	-9.6280	0.0032	0.0001
Neb	0.0000	0.3333	-9.6076	0.0147	0.0004
Gstz1	0.0000	4.5233	-9.6005	0.0000	0.0000
Pias3	0.0000	2.4367	-9.5765	0.0000	0.0000
Nfya	0.0000	1.8100	-9.5549	0.0002	0.0000
Slc35c2	0.0000	3.3000	-9.5026	0.0000	0.0000
Stil	0.0000	1.2300	-9.4701	0.0000	0.0000
Adgrl1	0.0000	0.8933	-9.4524	0.0000	0.0000
Gpr153	0.0000	1.5700	-9.4284	0.0000	0.0000
Nbr1	0.0000	1.3600	-9.3645	0.0000	0.0000
Rbfox2	0.0000	0.8633	-9.3611	0.0000	0.0000
Med14	0.0000	1.1733	-9.3539	0.0000	0.0000
3830406C13Rik	0.0000	2.5200	-9.3172	0.0000	0.0000
Wnt10b	0.0000	1.9533	-9.3099	0.0000	0.0000
Prkcsh	0.0000	2.8900	-9.2916	0.0222	0.0006
Kcnh2	0.0000	1.4067	-9.2240	0.0002	0.0000
Parn	0.0000	2.7167	-9.1899	0.0002	0.0000
Lnpk	0.0000	0.6400	-9.1897	0.0000	0.0000
Arhgap29	0.0000	1.0967	-9.1896	0.0034	0.0001
Slc25a13	0.0000	1.7100	-9.1492	0.0000	0.0000
Sema5a	0.0000	0.4767	-9.1439	0.0000	0.0000
Cand2	0.0000	1.4733	-9.1351	0.0000	0.0000
Amotl2	0.0000	1.2200	-9.0819	0.0000	0.0000
Tacc1	0.0000	0.6567	-9.0784	0.0000	0.0000
Eif5a	0.0000	3.6667	-9.0576	0.0000	0.0000
Dennd2a	0.0000	1.0567	-9.0540	0.0000	0.0000
Plxnb1	0.0000	0.5567	-9.0299	0.0000	0.0000
Nmt2	0.0000	1.0667	-8.9843	0.0000	0.0000
Cldnd1	0.0000	2.0900	-8.9717	0.0000	0.0000
Derl2	0.0000	1.2567	-8.9618	0.0000	0.0000
Gas2	0.0000	1.8833	-8.9573	0.0000	0.0000
Rnpc3	0.0000	1.1733	-8.9284	0.0000	0.0000
Iws1	0.0000	0.4533	-8.8810	0.0000	0.0000
Apbb2	0.0000	0.6867	-8.8701	0.0000	0.0000
Alg2	0.0000	1.4467	-8.8578	0.0000	0.0000
Pcnp	0.0000	1.8333	-8.8457	0.0000	0.0000
Frmd4a	0.0000	0.6133	-8.8325	0.0000	0.0000
Bub3	0.0000	2.0967	-8.8294	0.0000	0.0000
Fam160a1	0.0000	0.9667	-8.8294	0.0000	0.0000
Pkp4	0.0000	0.9067	-8.8155	0.0000	0.0000
Pan2	0.0000	0.9533	-8.8116	0.0000	0.0000
Gsn	0.0000	1.5300	-8.8106	0.0000	0.0000
Tank	0.0000	2.0700	-8.7675	0.0001	0.0000
Relch	0.0000	0.6767	-8.6610	0.0000	0.0000

Mgme1	0.0000	1.3233	-8.6327	0.0000	0.0000
Trim37	0.0000	1.0567	-8.5926	0.0000	0.0000
C1qtnf1	0.0000	0.8100	-8.5740	0.0000	0.0000
Zfp462	0.0000	0.3100	-8.5518	0.0044	0.0001
Phc2	0.0000	1.4467	-8.5340	0.0000	0.0000
Nav2	0.0000	0.4233	-8.5237	0.0000	0.0000
Ppfia1	0.0000	0.6267	-8.4540	0.0000	0.0000
Jsrp1	0.0433	10.8967	-8.4421	0.0070	0.0002
Hoxc4	0.0000	0.9233	-8.4364	0.0000	0.0000
Myo9b	0.0067	1.4700	-8.4217	0.0000	0.0000
Begain	0.0000	1.1700	-8.4165	0.0000	0.0000
Nol4l	0.0033	0.4867	-8.4158	0.0000	0.0000
Gab1	0.0000	0.7533	-8.3836	0.0002	0.0000
Tmem87b	0.0000	0.6333	-8.3535	0.0000	0.0000
Greb1	0.0000	0.3667	-8.3391	0.0000	0.0000
Sun1	0.0000	0.7267	-8.3335	0.0001	0.0000
Cox18	0.0033	2.2700	-8.3318	0.0000	0.0000
Ift122	0.0167	3.6333	-8.3120	0.0000	0.0000
Med1	0.0000	1.1233	-8.3028	0.0005	0.0000
Clock	0.0000	0.2733	-8.2789	0.0029	0.0001
Iqce	0.0000	0.5133	-8.2555	0.0001	0.0000
Rnft2	0.0000	0.6400	-8.2520	0.0000	0.0000
Riox2	0.0000	2.1133	-8.2479	0.0000	0.0000
Tmem175	0.0000	1.4500	-8.2039	0.0000	0.0000
Ewsr1	0.0300	7.6300	-8.1952	0.0000	0.0000
Gata6	0.0000	0.8700	-8.1760	0.0000	0.0000
Trappc12	0.0000	1.1967	-8.1742	0.0000	0.0000
Tbc1d23	0.0000	0.6733	-8.1082	0.0000	0.0000
Cyfip1	0.0000	0.9267	-8.0678	0.0000	0.0000
Zdhhc1	0.0000	1.0200	-8.0645	0.0084	0.0002
Ssbp4	0.0000	1.7200	-8.0331	0.0009	0.0000
Sema6c	0.0000	0.6000	-8.0182	0.0003	0.0000
Hnrnpc	0.0000	1.4533	-8.0180	0.0475	0.0014
Pbrm1	0.0000	0.3067	-8.0146	0.0354	0.0010
Zfp661	0.0000	0.3700	-7.9927	0.0000	0.0000
Nuggc	0.0033	0.9300	-7.9896	0.0006	0.0000
Mtmr11	0.0000	0.8600	-7.9861	0.0000	0.0000
Cep250	0.0100	1.5667	-7.9839	0.0000	0.0000
Itga7	0.0000	0.5633	-7.9648	0.0044	0.0001
Cep112	0.0000	0.6800	-7.9631	0.0268	0.0007
Cchcr1	0.0033	0.8100	-7.9437	0.0000	0.0000
Mtf2	0.0000	0.5467	-7.9414	0.0004	0.0000
Dcun1d2	0.0000	0.7167	-7.8792	0.0000	0.0000
Cnot3	0.0000	0.6267	-7.8705	0.0000	0.0000
Gprc5a	0.0000	1.0067	-7.8579	0.0000	0.0000
Ankrd54	0.0000	1.1400	-7.8406	0.0000	0.0000
Gtf2i	0.0000	0.3800	-7.8238	0.0000	0.0000
Phf21a	0.0000	0.4767	-7.7968	0.0000	0.0000
Armc9	0.0000	0.4367	-7.7835	0.0001	0.0000
Ebf3	0.0000	0.3867	-7.7753	0.0000	0.0000
Ermard	0.0000	0.7567	-7.7658	0.0000	0.0000
Dhx30	0.0000	0.4367	-7.7609	0.0007	0.0000
Srl	0.0000	0.5067	-7.7522	0.0036	0.0001
Ift74	0.0000	0.9167	-7.7485	0.0001	0.0000
Jarid2	0.0000	0.3600	-7.7364	0.0001	0.0000
Cgref1	0.0000	1.4933	-7.7357	0.0000	0.0000
Slx4ip	0.0000	0.3833	-7.7187	0.0000	0.0000
Zfp532	0.0033	0.3333	-7.6889	0.0000	0.0000
Rfx3	0.0000	0.2100	-7.6503	0.0008	0.0000
Ogdhl	0.0167	1.9633	-7.6181	0.0001	0.0000
Mindy3	0.0000	1.0600	-7.6149	0.0000	0.0000
Ppip5k1	0.0000	0.3067	-7.6008	0.0000	0.0000
Prpsap2	0.0100	1.1133	-7.5772	0.0001	0.0000
Zfp707	0.0000	0.9667	-7.5682	0.0000	0.0000
Nrip1	0.0000	0.3733	-7.5432	0.0010	0.0000
Ncam1	0.0000	0.3267	-7.5194	0.0000	0.0000
Lhx6	0.0000	0.5233	-7.5162	0.0000	0.0000
Urgcp	0.0000	0.4967	-7.5131	0.0045	0.0001
Csgalnact1	0.0000	0.4000	-7.4947	0.0001	0.0000
Rapgef3	0.0000	0.4200	-7.4776	0.0004	0.0000
Kctd9	0.0000	1.1000	-7.4684	0.0000	0.0000
Ablim1	0.0000	0.2567	-7.4610	0.0102	0.0002
Fggy	0.0000	0.9967	-7.4237	0.0025	0.0001
Lzts3	0.0000	0.3667	-7.4214	0.0000	0.0000
Rcor3	0.0000	0.7767	-7.4208	0.0002	0.0000

Fam189a1	0.0000	0.3267	-7.4169	0.0000	0.0000
Sema3f	0.0000	0.4867	-7.3751	0.0163	0.0004
Utrn	0.0100	1.6333	-7.3549	0.0002	0.0000
Ift81	0.0000	0.4867	-7.3508	0.0000	0.0000
Kctd17	0.0000	0.9300	-7.3353	0.0018	0.0000
Eno1	0.0000	0.8333	-7.3235	0.0002	0.0000
Nrap	0.0200	3.1367	-7.3024	0.0411	0.0012
Rhbdf1	0.0000	0.5100	-7.2966	0.0001	0.0000
Lrsam1	0.0000	0.3533	-7.2801	0.0000	0.0000
Gfod2	0.0000	0.3433	-7.2711	0.0002	0.0000
Hsf4	0.0000	0.8867	-7.2631	0.0001	0.0000
Epb41l1	0.0000	0.1633	-7.2487	0.0000	0.0000
Sox5	0.0000	0.1900	-7.2221	0.0027	0.0001
Arntl2	0.0000	0.4800	-7.1488	0.0001	0.0000
Cdc42bpa	0.0000	0.1533	-7.1193	0.0077	0.0002
Slc19a1	0.0000	0.6200	-7.1179	0.0008	0.0000
Postn	0.0000	0.4267	-7.1013	0.0001	0.0000
Casq1	0.3967	52.6800	-7.0896	0.0367	0.0010
Tdrkh	0.0000	0.3167	-7.0799	0.0006	0.0000
Baz2b	0.0000	0.1533	-7.0710	0.0000	0.0000
Lpgat1	0.0000	0.1867	-7.0530	0.0003	0.0000
Klc4	0.0000	0.5000	-7.0446	0.0034	0.0001
Myh1	0.3300	40.8767	-7.0325	0.0329	0.0009
Slc29a2	0.0000	0.9767	-7.0082	0.0001	0.0000
Fam110b	0.0000	0.3533	-7.0061	0.0015	0.0000
Slain2	0.0267	3.2533	-7.0016	0.0003	0.0000
Tcf7l2	0.0000	0.2967	-6.9947	0.0005	0.0000
Palm	0.0467	5.2000	-6.9625	0.0003	0.0000
Plppr2	0.0000	0.4500	-6.9547	0.0259	0.0007
Pde4b	0.0000	0.2900	-6.9486	0.0002	0.0000
Ces1a	0.0000	0.5800	-6.9464	0.0002	0.0000
Sprtn	0.0000	0.5700	-6.9155	0.0349	0.0010
Slc9a3r2	0.0000	0.5400	-6.8952	0.0001	0.0000
Pex2	0.0000	0.5133	-6.8858	0.0002	0.0000
Gpr173	0.0000	0.2033	-6.8371	0.0027	0.0001
St7	0.0000	0.4800	-6.7806	0.0003	0.0000
Acacb	0.0100	0.8167	-6.7539	0.0001	0.0000
Adamts16	0.0000	0.2033	-6.7344	0.0003	0.0000
Retreg3	0.0000	0.3133	-6.6777	0.0011	0.0000
Iqck	0.0000	0.6333	-6.6769	0.0397	0.0011
Slc41a3	0.0000	0.4133	-6.6062	0.0011	0.0000
Galr2	0.0000	0.3800	-6.5958	0.0241	0.0006
Rtkn	0.0133	0.7367	-6.5677	0.0070	0.0002
Aplp2	0.1067	8.7467	-6.5389	0.0244	0.0006
Bpnt1	0.0000	0.2200	-6.5255	0.0014	0.0000
Tmem209	0.0000	0.2600	-6.5252	0.0092	0.0002
Cdkl3	0.0000	0.2633	-6.5086	0.0010	0.0000
Dnm1	0.0367	3.2267	-6.4940	0.0059	0.0001
Mta1	0.1100	8.4367	-6.4815	0.0134	0.0003
Gng4	0.0000	0.2767	-6.4633	0.0167	0.0004
Dlgap1	0.0033	0.1533	-6.4122	0.0030	0.0001
Cep164	0.0000	0.1000	-6.3605	0.0072	0.0002
Aktip	0.0233	1.8667	-6.3561	0.0008	0.0000
Espn	0.0000	0.2167	-6.3446	0.0206	0.0005
Artn	0.0000	0.3367	-6.3101	0.0275	0.0007
Shroom2	0.0000	0.0933	-6.2503	0.0051	0.0001
Slc23a3	0.0000	0.2367	-6.2325	0.0125	0.0003
Sdk1	0.0000	0.0800	-6.2170	0.0113	0.0003
Apopt1	0.0467	2.3267	-6.2047	0.0008	0.0000
Lca5l	0.0000	0.2200	-6.1608	0.0160	0.0004
Cradd	0.0000	0.3867	-6.1572	0.0167	0.0004
Ralgapa2	0.0000	0.0767	-6.1444	0.0051	0.0001
Pax3	0.0000	0.1767	-6.1246	0.0189	0.0005
Bdh2	0.0000	0.6033	-6.1129	0.0185	0.0005
Kifc3	0.0000	0.1700	-6.0003	0.0097	0.0002
Celf2	0.0067	0.4400	-5.9954	0.0021	0.0000
Fam118b	0.0333	1.5633	-5.9767	0.0042	0.0001
Mgl2	0.0167	0.8500	-5.9425	0.0346	0.0010
Actr5	0.0000	0.2700	-5.9368	0.0463	0.0014
Ankrd29	0.0000	0.1667	-5.9194	0.0135	0.0003
Cdh23	0.0033	0.1033	-5.9063	0.0018	0.0000
Slco1a5	0.0000	0.2167	-5.9059	0.0279	0.0007
Cept1	0.0000	0.5867	-5.8533	0.0409	0.0012
Coq6	0.0000	0.3400	-5.8177	0.0342	0.0009
Lilrb4a	0.1133	6.1100	-5.8106	0.0330	0.0009
Daxx	0.1700	8.7933	-5.7575	0.0026	0.0001
Lmbr1	0.0100	0.4767	-5.7461	0.0382	0.0011

Paqr7	0.0000	0.1300	-5.7003	0.0330	0.0009
Tnni1	0.0233	0.7033	-5.5850	0.0046	0.0001
Kansl2	0.0133	0.4600	-5.5281	0.0107	0.0002
Mical2	0.0067	0.1867	-5.2776	0.0108	0.0002
Zfp637	0.0600	1.5733	-5.2629	0.0001	0.0000
Chga	0.0200	0.6367	-5.1979	0.0139	0.0003
Accs	0.0067	0.1767	-5.1442	0.0158	0.0004
Disp2	0.0100	0.3033	-5.0106	0.0299	0.0008
Pdzd7	0.0233	0.5933	-4.9662	0.0106	0.0002
Crlf1	0.1867	4.7733	-4.7109	0.0000	0.0000
Wdr37	0.1033	2.2000	-4.5325	0.0244	0.0006
Tmem38a	0.6133	13.4267	-4.4784	0.0322	0.0009
Nptx1	0.1133	2.1567	-4.3996	0.0343	0.0009
Dio3	0.0467	0.8167	-4.2275	0.0071	0.0002
Hspb6	5.9767	109.7467	-4.2216	0.0483	0.0014
Jmjd6	0.0667	1.0900	-4.1416	0.0005	0.0000
Pabpc1	5.0633	73.2767	-3.8961	0.0281	0.0007
Lyve1	0.2600	3.5400	-3.8299	0.0002	0.0000
Lmo7	0.0267	0.3267	-3.7031	0.0021	0.0000
Fbxl2	0.0667	0.5300	-3.4169	0.0424	0.0012
Hipk4	0.0767	0.6833	-3.3260	0.0356	0.0010
Cilp2	0.0433	0.4333	-3.2941	0.0043	0.0001
Arhgef10	0.3467	3.1833	-3.2736	0.0000	0.0000
Pgf	0.4467	3.7600	-3.1946	0.0179	0.0004
Atp5c1	2.6167	22.3533	-3.1067	0.0140	0.0003
Lox	0.7100	5.7467	-3.0895	0.0087	0.0002
Actn4	1.0367	8.0833	-3.0384	0.0082	0.0002
Nov	0.3367	2.4967	-2.9749	0.0019	0.0000
Ptprs	2.0033	14.0333	-2.8902	0.0001	0.0000
Gm10698	2.0000	13.7033	-2.8817	0.0016	0.0000
Ptk2	0.1933	1.2067	-2.7302	0.0011	0.0000
Serpinf1	14.2200	85.0600	-2.6673	0.0361	0.0010
Mgat3	1.0333	6.2467	-2.6516	0.0000	0.0000
Adar	0.9500	5.7167	-2.6499	0.0000	0.0000
Ctsk	2.5567	15.7700	-2.6358	0.0430	0.0012
Col18a1	0.2067	1.1833	-2.6333	0.0026	0.0001
Wbscr17	0.1267	0.6967	-2.5406	0.0219	0.0006
Vegfa	2.4300	13.1033	-2.4485	0.0210	0.0005
Wls	1.8333	9.3767	-2.4324	0.0098	0.0002
Mest	1.2133	6.3200	-2.4125	0.0236	0.0006
Gtf2ird1	0.2933	1.4500	-2.4001	0.0019	0.0000
Znhit1	1.1533	5.8900	-2.3934	0.0022	0.0000
Mfap5	2.6733	13.4500	-2.3858	0.0004	0.0000
Pllp	0.2433	1.2100	-2.3762	0.0131	0.0003
Col1a1	37.5200	184.1433	-2.3635	0.0001	0.0000
Cilp	3.1933	16.4367	-2.3458	0.0014	0.0000
Pi16	2.5633	12.5900	-2.2992	0.0186	0.0005
Tle1	0.4467	2.0467	-2.2949	0.0043	0.0001
Bcs1l	0.8133	3.6567	-2.2521	0.0000	0.0000
Eri3	4.1333	18.5067	-2.2089	0.0000	0.0000
Mmp19	0.5800	2.5833	-2.2087	0.0102	0.0002
Adamts8	1.0467	4.6133	-2.1866	0.0442	0.0013
Zfp687	0.7067	3.0700	-2.1726	0.0000	0.0000
Mgp	9.6500	42.4600	-2.1189	0.0475	0.0014
Ebf4	0.3000	1.2600	-2.0960	0.0033	0.0001
D630003M21Rik	0.2767	1.1100	-2.0699	0.0249	0.0006
Zfp428	0.8867	3.5733	-2.0683	0.0112	0.0003
Sec31a	0.5200	1.9700	-1.9886	0.0062	0.0001
Slc29a1	6.6867	25.1733	-1.9788	0.0001	0.0000
Plekha5	0.2667	1.0100	-1.9457	0.0034	0.0001
Gnas	1.5433	5.6067	-1.9206	0.0006	0.0000
Adck1	0.3667	1.3000	-1.9096	0.0091	0.0002
Adam12	0.4200	1.5000	-1.8750	0.0000	0.0000
Ncstn	3.9767	13.5767	-1.8352	0.0196	0.0005
Phf19	1.2467	4.2067	-1.8269	0.0103	0.0002
Mmp9	8.1233	28.9533	-1.8244	0.0281	0.0007
Pgghg	0.2400	0.8100	-1.8229	0.0115	0.0003
Sec24c	0.3633	1.2267	-1.8118	0.0020	0.0000
Col1a2	65.2533	217.0767	-1.8033	0.0405	0.0012
Cacna1a	0.1800	0.6167	-1.8022	0.0072	0.0002
Smyd2	2.6867	8.9500	-1.7647	0.0000	0.0000
Lypla1	0.5133	1.6433	-1.7535	0.0370	0.0010
Gabbr1	0.7167	2.3600	-1.7146	0.0299	0.0008
Loxl1	10.9433	33.6733	-1.6800	0.0156	0.0004
Tmem65	0.2367	0.7333	-1.6774	0.0350	0.0010
Acad8	1.0800	3.2567	-1.6579	0.0288	0.0008
Tead3	3.2800	9.9200	-1.6561	0.0295	0.0008
Ncor2	1.3967	4.2533	-1.6387	0.0000	0.0000
Sgms2	0.3367	0.9867	-1.5931	0.0081	0.0002
Akt1s1	1.7767	5.2833	-1.5905	0.0000	0.0000
Ddah1	0.4033	1.1700	-1.5893	0.0234	0.0006
Pkd1	0.4833	1.3867	-1.5889	0.0341	0.0009

Thbs2	2.9267	8.4233	-1.5748	0.0006	0.0000
Pheta1	1.5700	4.4833	-1.5692	0.0106	0.0002
Pdxdc1	1.1333	3.1967	-1.5462	0.0017	0.0000
Brf2	1.4500	4.1500	-1.5450	0.0030	0.0001
Dlg4	1.8467	5.1233	-1.5449	0.0370	0.0010
C1qtnf6	2.6867	7.5133	-1.5417	0.0386	0.0011
Paip2	2.0500	5.6733	-1.5375	0.0363	0.0010
Rftn2	0.5900	1.6900	-1.5215	0.0397	0.0011
Clk2	2.1033	5.9333	-1.5207	0.0033	0.0001
Agap3	2.0867	5.7500	-1.5108	0.0085	0.0002
Mvk	3.6800	9.8267	-1.4886	0.0348	0.0010
Prpf19	3.1467	8.4267	-1.4814	0.0179	0.0004
St3gal4	4.7667	12.7567	-1.4703	0.0228	0.0006
Stra6	4.6233	12.4700	-1.4614	0.0458	0.0013
Ubtd1	10.9300	29.1400	-1.4536	0.0022	0.0000
Rptor	2.7800	7.0767	-1.4094	0.0020	0.0000
Zfp629	0.8933	2.2400	-1.3736	0.0433	0.0013
Ndufa4l2	3.4400	8.4267	-1.3351	0.0089	0.0002
Plekha6	1.8033	4.3733	-1.3138	0.0249	0.0006
Tmem218	1.1300	2.7333	-1.2998	0.0297	0.0008
Arntl	3.8100	9.1133	-1.2984	0.0001	0.0000
Klc1	2.2467	5.4367	-1.2927	0.0397	0.0011
Slit3	1.6100	3.8567	-1.2925	0.0000	0.0000
Map1a	0.5400	1.2967	-1.2870	0.0048	0.0001
Prmt2	1.2300	2.8867	-1.2817	0.0397	0.0011
H2afy	4.7967	11.2700	-1.2726	0.0120	0.0003
Adam8	7.0933	16.3600	-1.2530	0.0039	0.0001
Agpat2	3.4133	7.8333	-1.2371	0.0121	0.0003
Gale	4.3667	9.8733	-1.2180	0.0015	0.0000
Dtx4	1.1167	2.4967	-1.2127	0.0003	0.0000
Adm	2.2033	4.9367	-1.1965	0.0091	0.0002
2310039H08Rik	18.2933	40.2900	-1.1887	0.0281	0.0007
Hcfc1r1	37.4933	82.9700	-1.1809	0.0069	0.0002
Ift140	2.2500	4.8933	-1.1694	0.0143	0.0003
Zmynd11	1.1533	2.5333	-1.1564	0.0010	0.0000
Ube2e2	4.1100	8.8400	-1.1431	0.0218	0.0005
Rnf25	7.0333	15.0133	-1.1341	0.0002	0.0000
Mindy1	4.6933	10.0233	-1.1250	0.0498	0.0015
Pcbp2	17.4467	36.8267	-1.1158	0.0000	0.0000
Nid2	2.0133	4.2233	-1.1114	0.0415	0.0012
Abl2	1.2500	2.6200	-1.1070	0.0423	0.0012
Slc6a6	0.6000	1.2500	-1.0937	0.0012	0.0000
Mtch1	30.2767	62.9100	-1.0876	0.0286	0.0008
Rhoj	8.0800	16.8033	-1.0840	0.0384	0.0011
Nufip2	0.9533	1.9567	-1.0818	0.0223	0.0006
Tnk2	2.9567	6.0833	-1.0793	0.0123	0.0003
Hist1h1c	30.4700	62.9967	-1.0732	0.0001	0.0000
Tpm3	17.2667	35.6633	-1.0618	0.0003	0.0000
Tbc1d20	0.9900	2.0300	-1.0602	0.0199	0.0005
Igfbp3	47.4967	96.5200	-1.0552	0.0002	0.0000
Tada3	3.5500	7.0400	-1.0252	0.0311	0.0008
Micall2	6.9000	13.4633	-1.0004	0.0176	0.0004
Flcn	5.0033	9.7133	-1.0003	0.0295	0.0008
Gapdh-ps15	19.2767	37.7433	-0.9900	0.0006	0.0000
Ctgf	11.9700	23.2100	-0.9893	0.0097	0.0002
Dcaf8	5.9133	11.5433	-0.9781	0.0032	0.0001
Plpp3	9.5033	17.9433	-0.9435	0.0295	0.0008
Gtf3c5	6.7200	12.2667	-0.9084	0.0039	0.0001
Tbc1d9b	1.3700	2.5033	-0.9064	0.0023	0.0000
2310009B15Rik	7.4933	13.6433	-0.8875	0.0268	0.0007
Tpcn1	28.1667	49.7500	-0.8663	0.0146	0.0003
Tnrc18	3.1600	5.5367	-0.8434	0.0061	0.0001
Cebpzos	3.9267	6.8600	-0.8310	0.0207	0.0005
Eva1b	12.6733	21.7967	-0.8139	0.0314	0.0008
Mrnip	4.1867	7.0767	-0.7903	0.0310	0.0008
Sirt6	8.9900	15.0700	-0.7743	0.0295	0.0008
Ccdc102a	6.5700	10.9800	-0.7699	0.0475	0.0014
Tgfbr3	3.9167	6.4700	-0.7538	0.0337	0.0009
Ubap2l	2.0033	3.2567	-0.7289	0.0239	0.0006
Kdm4a	10.5267	16.8333	-0.7140	0.0087	0.0002
4931406P16Rik	5.6567	9.0700	-0.7123	0.0035	0.0001
Ptov1	3.4667	5.3867	-0.6588	0.0085	0.0002
BC004004	17.9133	27.2867	-0.6370	0.0249	0.0006
Tmem138	11.1033	15.7233	-0.5300	0.0204	0.0005
Stk38	9.4633	6.0600	0.6290	0.0391	0.0011
Pin4	33.7333	20.9233	0.6646	0.0201	0.0005
Mapk14	14.2767	8.6667	0.7013	0.0494	0.0015
LOC108167440	78.1100	46.7133	0.7109	0.0088	0.0002
Psmd5	7.7967	4.6767	0.7236	0.0092	0.0002
Prps2	12.7467	7.5767	0.7381	0.0072	0.0002
Ctsc	24.9300	14.4500	0.7598	0.0000	0.0000
C9orf72	4.5000	2.5267	0.8047	0.0420	0.0012

Wac	1.8300	1.0200	0.8285	0.0399	0.0011
Zscan29	2.6400	1.4600	0.8457	0.0360	0.0010
Snx11	5.7633	2.9800	0.9356	0.0003	0.0000
Usp37	3.2200	1.5867	0.9820	0.0078	0.0002
Lyz2	1069.9700	533.6667	0.9892	0.0005	0.0000
Tmc4	3.4000	1.6633	1.0066	0.0021	0.0000
Cgas	3.7200	1.8433	1.0083	0.0221	0.0006
BC017158	7.4400	3.6767	1.0105	0.0224	0.0006
Ctss	212.9200	102.0167	1.0191	0.0005	0.0000
Stat5b	1.3167	0.6400	1.0229	0.0088	0.0002
Atg5	11.3367	5.5100	1.0399	0.0471	0.0014
Lyz1	99.0367	47.2233	1.0612	0.0241	0.0006
Safb2	3.6200	1.7100	1.0627	0.0423	0.0012
Cab39l	12.9167	6.1200	1.0698	0.0198	0.0005
Zcchc8	1.2267	0.5633	1.0916	0.0077	0.0002
Tial1	26.0267	11.7067	1.1282	0.0485	0.0014
Inpp1	2.1200	0.9533	1.1348	0.0055	0.0001
Ogt	12.8000	5.7233	1.1424	0.0085	0.0002
Xrn1	1.3400	0.5933	1.1568	0.0009	0.0000
Clasp2	1.2233	0.5300	1.1593	0.0310	0.0008
Tlr8	1.2467	0.5400	1.1864	0.0007	0.0000
Pttg1	3.6633	1.5800	1.2154	0.0083	0.0002
Csf2ra	30.8700	13.2467	1.2165	0.0268	0.0007
Erap1	9.5133	4.0100	1.2375	0.0002	0.0000
Nabp1	8.9167	3.7600	1.2460	0.0167	0.0004
Tnfsf13	3.8300	1.5767	1.2575	0.0026	0.0001
Zbtb18	2.9333	1.2133	1.2634	0.0078	0.0002
Cd84	8.8933	3.6367	1.2721	0.0478	0.0014
Tor1aip2	2.2800	0.9400	1.2807	0.0372	0.0010
Reep6	6.4433	2.6200	1.2922	0.0030	0.0001
Omp	1.4733	0.5867	1.2960	0.0191	0.0005
Poc5	1.9300	0.7800	1.2961	0.0000	0.0000
Clstn3	0.6900	0.2733	1.3138	0.0154	0.0004
Wdr20	0.7367	0.2900	1.3282	0.0388	0.0011
Cd274	16.1633	6.3667	1.3289	0.0104	0.0002
Mphosph9	2.2867	0.9067	1.3299	0.0147	0.0004
Nlrc5	10.4700	4.1000	1.3430	0.0005	0.0000
Cp	4.5733	1.7900	1.3487	0.0012	0.0000
Nmb	0.4233	0.1633	1.3569	0.0399	0.0011
Tgtp2	65.1033	24.8700	1.3842	0.0270	0.0007
Cxcl9	48.6400	18.3300	1.3950	0.0364	0.0010
Irgm1	63.9633	23.9567	1.4074	0.0123	0.0003
217615	8.4967	3.1733	1.4200	0.0178	0.0004
Parp14	28.7267	10.7200	1.4314	0.0251	0.0007
Ipmk	6.4533	2.3000	1.4485	0.0167	0.0004
Carnmt1	1.9333	0.6900	1.4563	0.0417	0.0012
Gbp5	23.4700	8.5100	1.4609	0.0035	0.0001
Usp38	6.1333	2.1633	1.5100	0.0228	0.0006
Gm4841	6.0033	2.0567	1.5114	0.0064	0.0001
Optn	3.8067	1.2933	1.5444	0.0008	0.0000
Gm12185	3.6100	1.2433	1.5505	0.0231	0.0006
Cox17	3.5833	1.2367	1.5519	0.0288	0.0008
Atp8b4	2.4967	0.8500	1.5586	0.0381	0.0011
Abcc5	1.3633	0.4600	1.5624	0.0144	0.0003
Mycbp2	0.3633	0.1233	1.5745	0.0023	0.0000
Wiz	2.7967	0.9000	1.6145	0.0040	0.0001
Psme2	5.8533	1.8367	1.6500	0.0237	0.0006
Ccnl1	1.5700	0.4933	1.6501	0.0169	0.0004
Serpinb6b	18.5767	5.8967	1.6668	0.0414	0.0012
Tlcd2	4.1333	1.3033	1.6734	0.0071	0.0002
Itsn2	6.4000	1.9833	1.6965	0.0451	0.0013
Gclc	15.0067	4.6400	1.6995	0.0375	0.0010
Tom1l1	3.4933	1.0700	1.7025	0.0000	0.0000
Dgki	0.1833	0.0567	1.7046	0.0466	0.0014
Herc2	3.0200	0.9000	1.7047	0.0092	0.0002
Gm12250	24.6167	7.5467	1.7078	0.0064	0.0001
Zfp672	1.6100	0.4733	1.7321	0.0005	0.0000
Rhbdf2	8.4067	2.4967	1.7571	0.0241	0.0006
Malt1	7.5267	2.2067	1.7711	0.0314	0.0008
Ctrl	2.0333	0.5900	1.7751	0.0314	0.0008
Fyb	3.4133	0.9867	1.7815	0.0397	0.0011
Gata3	3.3733	0.9633	1.8018	0.0000	0.0000
Mcmdc2	3.2500	0.9367	1.8106	0.0172	0.0004
Usp48	3.8567	1.0967	1.8249	0.0245	0.0006
Klrc1	2.7667	0.7733	1.8380	0.0441	0.0013
Klra2	4.0300	1.0967	1.8584	0.0000	0.0000
D1Ertd622e	5.0333	1.3967	1.8773	0.0361	0.0010
Zfp983	1.0300	0.2733	1.8985	0.0337	0.0009
Rbm3	13.4400	3.5200	1.9075	0.0076	0.0002
Pum2	2.8500	0.7400	1.9153	0.0043	0.0001
Il1a	2.4800	0.6333	1.9415	0.0001	0.0000
4930523C07Rik	0.9433	0.2400	1.9437	0.0001	0.0000

Clec12a	2.4433	0.6267	1.9817	0.0016	0.0000
Ppef2	0.6167	0.1667	1.9820	0.0471	0.0014
Zbtb32	3.9500	0.9433	2.0257	0.0174	0.0004
Far2	0.9133	0.2233	2.0369	0.0384	0.0011
Pnpt1	5.3267	1.2600	2.0640	0.0013	0.0000
Il18rap	4.0367	0.9833	2.0697	0.0008	0.0000
Sirt7	2.4233	0.5900	2.0704	0.0226	0.0006
1700066C05Rik	3.4033	0.8333	2.0806	0.0142	0.0003
Ltb4r2	1.0667	0.2600	2.0823	0.0441	0.0013
Lmbrd1	2.7767	0.6567	2.0837	0.0093	0.0002
Chmp2a	4.6133	1.0333	2.0955	0.0184	0.0005
Fam111a	1.6733	0.3933	2.1340	0.0358	0.0010
Edem1	3.3167	0.7567	2.1395	0.0420	0.0012
Lcp1	2.0900	0.4800	2.1539	0.0335	0.0009
Gbp4	18.1367	4.0467	2.1849	0.0000	0.0000
Dmxl1	1.3967	0.3033	2.2032	0.0131	0.0003
Casp8	8.3700	1.8033	2.2060	0.0228	0.0006
Cdk12	2.5500	0.5433	2.2147	0.0228	0.0006
Ppm1h	1.9233	0.4233	2.2147	0.0437	0.0013
Tnk1	0.4500	0.1000	2.2207	0.0355	0.0010
Tmem51	4.7367	1.0133	2.2441	0.0329	0.0009
Man1b1	9.3733	1.8467	2.3181	0.0000	0.0000
Cybb	42.2433	8.5700	2.3287	0.0185	0.0005
Gbp9	7.5033	1.4733	2.3752	0.0089	0.0002
Zfp207	2.9433	0.5433	2.4595	0.0389	0.0011
Alx4	0.5867	0.1100	2.4648	0.0491	0.0015
Kmt2c	0.3967	0.0667	2.4683	0.0092	0.0002
Gda	9.8300	1.7867	2.4805	0.0243	0.0006
Rbl1	5.9200	1.0233	2.5474	0.0008	0.0000
Ncor1	1.0600	0.1800	2.5511	0.0006	0.0000
Pstpip1	7.1700	1.2300	2.5559	0.0019	0.0000
Dennd4a	7.9767	1.3667	2.5640	0.0340	0.0009
Zbtb8os	1.8200	0.3467	2.5644	0.0074	0.0002
Ifi203	6.7033	1.1167	2.5914	0.0366	0.0010
Fam213a	4.1100	0.7067	2.6187	0.0079	0.0002
Mdm4	11.1933	1.8067	2.6233	0.0021	0.0000
Ehbp1l1	5.1333	0.8333	2.6561	0.0100	0.0002
Dennd6a	0.7933	0.1267	2.6733	0.0228	0.0006
Vav3	1.0167	0.1633	2.6898	0.0278	0.0007
Dkkl1	4.6733	0.7133	2.7484	0.0235	0.0006
Trim30a	38.2900	5.6700	2.8011	0.0246	0.0006
Cpox	86.5933	11.9933	2.8536	0.0340	0.0009
Plbd1	2.7867	0.4033	2.8680	0.0430	0.0012
Asb13	3.7300	0.5033	2.9042	0.0061	0.0001
H2-M2	2.0267	0.2767	2.9238	0.0046	0.0001
Plcl2	2.7567	0.3733	2.9384	0.0363	0.0010
Tfrc	38.4700	5.0367	2.9441	0.0091	0.0002
Cd244a	3.2267	0.4233	2.9670	0.0130	0.0003
Galnt3	1.5467	0.2100	2.9682	0.0369	0.0010
Pou6f1	2.2367	0.2967	2.9847	0.0373	0.0010
Jaml	5.1467	0.6733	2.9934	0.0332	0.0009
Pik3r5	8.5467	1.0367	3.0714	0.0162	0.0004
Lrrc75b	0.5633	0.0733	3.0779	0.0204	0.0005
Fam241a	20.2433	2.3833	3.1064	0.0199	0.0005
Baz1a	2.3167	0.2667	3.1082	0.0295	0.0008
Gvin1	2.1800	0.2400	3.1251	0.0342	0.0009
Slfn8	2.9233	0.3333	3.1660	0.0000	0.0000
Gpr68	1.0967	0.1333	3.1736	0.0019	0.0000
Pilrb1	9.9933	1.1200	3.1854	0.0303	0.0008
Metap2	22.7600	2.5300	3.1885	0.0008	0.0000
Gbp8	11.9433	1.3467	3.1927	0.0031	0.0001
Fam84a	0.6967	0.0733	3.2058	0.0407	0.0012
Npnt	8.2733	0.9100	3.2224	0.0370	0.0010
Serpina3f	9.0133	0.9900	3.2252	0.0037	0.0001
Entpd3	0.3000	0.0300	3.2259	0.0460	0.0013
Sh3bp2	4.8100	0.5367	3.2276	0.0128	0.0003
Acmsd	1.8767	0.2167	3.2288	0.0295	0.0008
Efhd1	5.2900	0.5767	3.2671	0.0424	0.0012
Slc25a37	113.2133	11.7367	3.2726	0.0279	0.0007
Rnf123	2.7300	0.2900	3.2879	0.0335	0.0009
Dennd2d	5.0833	0.5333	3.3282	0.0487	0.0014
Mef2c	2.4067	0.2367	3.3623	0.0225	0.0006
Mapk13	1.6867	0.1633	3.3693	0.0053	0.0001
Gypc	5.4033	0.5300	3.3832	0.0197	0.0005
Tmem26	2.9400	0.2867	3.3893	0.0270	0.0007
Sirpa	17.6933	1.6067	3.4593	0.0089	0.0002
Grk3	4.6567	0.4267	3.5268	0.0139	0.0003
F2rl2	3.9100	0.3500	3.5314	0.0373	0.0010
Fam49a	6.4967	0.5800	3.5480	0.0363	0.0010
Cep70	0.6200	0.0600	3.5596	0.0120	0.0003
Acot1	1.6367	0.1467	3.5617	0.0002	0.0000
Gca	2.2567	0.1967	3.5690	0.0470	0.0014

Epb41	11.6033	1.0000	3.5722	0.0192	0.0005
Ear2	13.1800	1.1233	3.5761	0.0237	0.0006
Slc46a3	5.6700	0.5033	3.5776	0.0370	0.0010
Rasal2	0.7767	0.0600	3.5922	0.0035	0.0001
Trmo	1.8767	0.1567	3.6337	0.0258	0.0007
Iqgap2	4.9667	0.4100	3.6458	0.0258	0.0007
Taf10	2.5633	0.2133	3.6648	0.0423	0.0012
Ezh2	8.4700	0.6233	3.6659	0.0397	0.0011
Ces2g	10.5433	0.8600	3.6705	0.0371	0.0010
Adrb1	1.2767	0.1033	3.6729	0.0292	0.0008
Slc39a8	3.2300	0.2600	3.7180	0.0089	0.0002
Mkrn1	18.7667	1.4233	3.7265	0.0334	0.0009
Pilrb2	2.0967	0.1567	3.7768	0.0108	0.0002
Fpr1	4.8367	0.3433	3.8590	0.0064	0.0001
Epor	17.9833	1.2167	3.9383	0.0393	0.0011
Sec14l2	9.6633	0.6267	3.9453	0.0115	0.0003
Ccdc125	1.1800	0.0833	3.9794	0.0285	0.0008
Ifit1bl1	7.2933	0.4900	3.9951	0.0423	0.0012
Gzma	53.5433	3.4300	4.0067	0.0139	0.0003
Timeless	0.1333	0.0100	4.0392	0.0121	0.0003
Lamc3	0.3133	0.0167	4.0666	0.0003	0.0000
Bpgm	73.8667	4.3900	4.1063	0.0151	0.0004
Dhrs11	21.8700	1.2867	4.1143	0.0193	0.0005
Ank1	1.0200	0.0533	4.2611	0.0170	0.0004
Siglece	16.5300	0.8700	4.3427	0.0234	0.0006
Dgkg	0.6067	0.0300	4.4208	0.0310	0.0008
Muc13	0.7500	0.0400	4.4248	0.0015	0.0000
Gp6	0.3633	0.0200	4.5082	0.0220	0.0006
Ache	6.3667	0.3000	4.5213	0.0177	0.0004
Kbtbd6	1.0967	0.0600	4.5303	0.0486	0.0014
Klrc3	1.0400	0.0500	4.5708	0.0377	0.0011
Cldn10	1.3433	0.0500	4.5862	0.0312	0.0008
Tent5c	39.7533	1.5933	4.6632	0.0142	0.0003
Akr1b7	1.3400	0.0533	4.6950	0.0237	0.0006
Ifitm6	20.6167	0.8100	4.7158	0.0356	0.0010
Ifnar2	6.4933	0.2300	4.7630	0.0333	0.0009
Abcg4	9.7500	0.3700	4.7654	0.0045	0.0001
Vipr1	1.1033	0.0433	4.8008	0.0073	0.0002
F5	1.4900	0.0533	4.8385	0.0110	0.0003
Clca3a1	7.9400	0.2767	4.8792	0.0179	0.0004
Erfe	2.2233	0.0800	4.9213	0.0299	0.0008
Ypel4	15.6367	0.5133	4.9557	0.0047	0.0001
Lcn2	77.1133	2.5767	4.9746	0.0011	0.0000
Atp7b	1.4033	0.0500	4.9763	0.0015	0.0000
Icam4	13.6600	0.4333	4.9962	0.0220	0.0006
Scn5a	0.1200	0.0067	5.0008	0.0401	0.0011
Ankrd12	0.7000	0.0233	5.0110	0.0089	0.0002
Mme	0.1400	0.0033	5.0516	0.0241	0.0006
S1pr5	2.0600	0.0667	5.0595	0.0147	0.0004
Slamf7	0.8167	0.0333	5.0972	0.0495	0.0015
Rhd	82.0033	2.3433	5.1142	0.0061	0.0001
Gm867	25.2267	0.7200	5.1808	0.0017	0.0000
Recql5	4.3933	0.1333	5.2006	0.0000	0.0000
Sorbs2	0.4167	0.0133	5.2473	0.0186	0.0005
Ptprc	15.4533	0.4133	5.2521	0.0140	0.0003
Cd24a	491.1833	13.2400	5.2622	0.0364	0.0010
Grb7	0.6433	0.0133	5.3016	0.0088	0.0002
Golph3l	5.2967	0.1400	5.3813	0.0000	0.0000
Tmem35a	4.4400	0.1100	5.3840	0.0041	0.0001
Plac8	0.9133	0.0333	5.4087	0.0238	0.0006
a	3.3533	0.0800	5.5450	0.0002	0.0000
Cfap74	0.1700	0.0067	5.5533	0.0270	0.0007
Cysltr2	0.9533	0.0267	5.5714	0.0134	0.0003
Slc40a1	20.8767	0.4500	5.5848	0.0114	0.0003
Prtn3	23.0233	0.4900	5.6242	0.0340	0.0009
Mfsd2b	21.5800	0.4433	5.6584	0.0014	0.0000
Lrrc27	0.3200	0.0000	5.6803	0.0372	0.0010
Dram1	0.3800	0.0000	5.7166	0.0455	0.0013
Map2	0.0900	0.0000	5.7187	0.0377	0.0011
Pde10a	0.0900	0.0033	5.7253	0.0435	0.0013
Srr	0.1567	0.0000	5.7360	0.0417	0.0012
Gdf6	2.5533	0.0500	5.7547	0.0191	0.0005
Cnot2	2.0667	0.0400	5.8078	0.0034	0.0001
Atrx	0.0567	0.0000	5.8089	0.0397	0.0011
Ddx6	0.2067	0.0000	5.8144	0.0377	0.0011
C130050O18Rik	0.3367	0.0000	5.8202	0.0266	0.0007
Isg20	14.9733	0.2933	5.8283	0.0064	0.0001
Dusp3	1.7300	0.0333	5.8592	0.0030	0.0001
Abcg2	2.4467	0.0500	5.8752	0.0151	0.0004
Capn3	0.7433	0.0133	5.8997	0.0268	0.0007
Xpo6	0.5133	0.0100	5.9124	0.0002	0.0000
2610528J11Rik	0.6300	0.0000	5.9822	0.0142	0.0003

Slc6a4	2.8067	0.0467	6.0000	0.0226	0.0006
Mboat2	0.4833	0.0067	6.0472	0.0311	0.0008
Chadl	0.2467	0.0000	6.0720	0.0123	0.0003
Cenpp	0.8767	0.0000	6.0863	0.0088	0.0002
Mterf2	0.3700	0.0000	6.0937	0.0080	0.0002
Zfp810	0.9700	0.0167	6.1187	0.0066	0.0001
Scamp1	0.1700	0.0000	6.1344	0.0073	0.0002
Ttc6	0.1933	0.0000	6.1608	0.0151	0.0004
Rhox8	0.5633	0.0000	6.1892	0.0417	0.0012
Phyhip	7.0500	0.0933	6.1961	0.0156	0.0004
Gfap	0.2867	0.0000	6.2147	0.0422	0.0012
Naa35	0.3433	0.0000	6.2324	0.0110	0.0003
Nr4a2	0.2233	0.0000	6.2370	0.0332	0.0009
Daglb	1.0567	0.0133	6.2484	0.0332	0.0009
Palb2	0.9400	0.0133	6.2618	0.0000	0.0000
Gp5	5.3467	0.0700	6.2642	0.0017	0.0000
Ptpn22	0.1967	0.0000	6.2852	0.0087	0.0002
Bend3	0.1167	0.0000	6.2854	0.0097	0.0002
Cblc	0.4300	0.0000	6.2932	0.0049	0.0001
Mmaa	0.1367	0.0000	6.2972	0.0064	0.0001
Wnk1	0.8367	0.0100	6.3087	0.0021	0.0000
Cenph	3.6467	0.0500	6.3092	0.0010	0.0000
Bcas1	0.2433	0.0000	6.3107	0.0088	0.0002
Dennd2c	0.1300	0.0000	6.3553	0.0165	0.0004
Shld2	0.2033	0.0000	6.3659	0.0035	0.0001
Dock9	0.0900	0.0000	6.3949	0.0176	0.0004
Fbxo47	0.1800	0.0000	6.4038	0.0025	0.0000
Tshz2	0.1633	0.0000	6.4053	0.0470	0.0014
Dmtn	0.9033	0.0133	6.4187	0.0214	0.0005
Ikzf1	3.4867	0.0433	6.4289	0.0413	0.0012
Abi3bp	0.1767	0.0000	6.4383	0.0340	0.0009
Pdzk1ip1	23.4033	0.2800	6.4420	0.0070	0.0002
Rnf14	0.2467	0.0000	6.4509	0.0014	0.0000
Pola2	2.2900	0.0333	6.4539	0.0000	0.0000
Irx4	0.3467	0.0000	6.4805	0.0168	0.0004
Clec4a4	1.4333	0.0200	6.4821	0.0283	0.0007
Thsd1	0.1867	0.0000	6.4898	0.0014	0.0000
Il3ra	0.5700	0.0000	6.4944	0.0017	0.0000
Tmem63c	0.1767	0.0000	6.4958	0.0202	0.0005
S100a8	201.5933	2.3600	6.4959	0.0010	0.0000
Lrrc56	0.4233	0.0000	6.5096	0.0197	0.0005
Gak	0.1867	0.0000	6.5671	0.0104	0.0002
Vrk2	0.5000	0.0000	6.5765	0.0018	0.0000
Sin3a	1.5067	0.0167	6.5805	0.0002	0.0000
Matk	0.4933	0.0000	6.6040	0.0015	0.0000
Fbxl4	0.4733	0.0000	6.6295	0.0016	0.0000
Ppwd1	0.5633	0.0000	6.6377	0.0073	0.0002
Me3	0.2300	0.0000	6.6526	0.0071	0.0002
Mtus1	0.1500	0.0000	6.6772	0.0070	0.0002
Gmfg	0.9567	0.0000	6.6811	0.0010	0.0000
Hgf	0.3433	0.0000	6.7099	0.0028	0.0001
Alas2	54.3967	0.5267	6.7258	0.0002	0.0000
Podxl2	0.4533	0.0000	6.7331	0.0159	0.0004
Nat14	0.6933	0.0000	6.7580	0.0111	0.0003
Shroom3	0.1433	0.0000	6.7625	0.0006	0.0000
Bzw1	5.2267	0.0500	6.7835	0.0028	0.0001
Fgd4	0.1200	0.0000	6.7911	0.0005	0.0000
Rfx5	0.1833	0.0000	6.7984	0.0004	0.0000
Fn3k	2.9833	0.0267	6.8100	0.0282	0.0007
As3mt	0.5567	0.0000	6.8498	0.0017	0.0000
Mylk3	2.1967	0.0167	6.8615	0.0440	0.0013
Stxbp5	0.3067	0.0000	6.9024	0.0031	0.0001
Samd11	3.1033	0.0300	6.9074	0.0241	0.0006
Trim30d	0.8667	0.0000	6.9257	0.0057	0.0001
Tspan33	22.5467	0.1800	6.9269	0.0046	0.0001
Enox2	0.2800	0.0000	6.9439	0.0003	0.0000
Ube2e3	0.5500	0.0000	6.9582	0.0010	0.0000
Zcchc9	0.8767	0.0000	6.9630	0.0032	0.0001
Blm	0.2367	0.0000	6.9633	0.0010	0.0000
Ttc41	0.1967	0.0000	6.9815	0.0039	0.0001
Igf1	0.1533	0.0000	6.9883	0.0005	0.0000
Nfe2	11.1833	0.0967	7.0493	0.0000	0.0000
Mpp7	0.2433	0.0000	7.0639	0.0070	0.0002
Mea1	1.2400	0.0000	7.1004	0.0231	0.0006
Zfp758	0.2967	0.0000	7.1069	0.0011	0.0000
Cdc6	0.2700	0.0000	7.1088	0.0001	0.0000
Csf3r	1.6967	0.0167	7.1655	0.0046	0.0001
Ammecr1l	0.2767	0.0000	7.1798	0.0045	0.0001
Zfp41	0.3433	0.0000	7.2020	0.0008	0.0000
Cadps2	0.2700	0.0000	7.2108	0.0007	0.0000
Nrip3	0.6500	0.0033	7.2270	0.0327	0.0009
Ift88	0.4700	0.0000	7.2320	0.0028	0.0001

Tgfb1i1	0.7900	0.0000	7.2440	0.0004	0.0000
Dpep2	1.0200	0.0000	7.2514	0.0008	0.0000
Slamf6	0.5400	0.0000	7.2551	0.0085	0.0002
B430306N03Rik	0.3967	0.0000	7.2566	0.0019	0.0000
Treml1	7.7067	0.0533	7.2630	0.0054	0.0001
Ikzf4	0.2600	0.0000	7.2706	0.0084	0.0002
Ly6g	2.9967	0.0200	7.2846	0.0293	0.0008
Mtrr	0.4333	0.0000	7.3221	0.0424	0.0012
St3gal6	0.8267	0.0000	7.3427	0.0022	0.0000
Col17a1	0.2700	0.0000	7.3443	0.0001	0.0000
Gsap	0.3933	0.0000	7.3596	0.0000	0.0000
Arid3b	0.3667	0.0000	7.3610	0.0000	0.0000
Sbno1	0.2700	0.0000	7.3694	0.0007	0.0000
Tmprss13	0.4533	0.0000	7.3939	0.0001	0.0000
Dlc1	0.2567	0.0000	7.4268	0.0155	0.0004
Il1rl2	0.3800	0.0000	7.4304	0.0002	0.0000
Itgad	8.4100	0.0567	7.4699	0.0093	0.0002
Ankle1	0.6100	0.0000	7.5299	0.0024	0.0000
Aqp9	0.6000	0.0000	7.5372	0.0009	0.0000
S100a9	184.1300	0.9967	7.5383	0.0064	0.0001
Tmem215	1.3967	0.0067	7.5493	0.0217	0.0005
Ppbp	80.8300	0.4367	7.5853	0.0009	0.0000
Zfp451	0.4433	0.0000	7.5945	0.0012	0.0000
Spag7	1.5933	0.0000	7.6146	0.0000	0.0000
Tal1	15.3767	0.0833	7.6368	0.0407	0.0012
Snca	8.1300	0.0400	7.6609	0.0024	0.0000
Fkrp	4.7133	0.0300	7.6650	0.0343	0.0009
Rasa4	0.6433	0.0000	7.6653	0.0001	0.0000
C1qtnf5	1.5033	0.0000	7.6723	0.0000	0.0000
Cenpi	0.5267	0.0000	7.6825	0.0002	0.0000
Ctse	156.5233	0.7767	7.7086	0.0020	0.0000
Pik3cd	2.0267	0.0100	7.7154	0.0000	0.0000
Ltf	35.7067	0.1667	7.7431	0.0000	0.0000
Slc38a9	0.2167	0.0000	7.7459	0.0009	0.0000
Ktn1	0.3000	0.0000	7.7460	0.0001	0.0000
Mtx3	1.1467	0.0067	7.7795	0.0000	0.0000
Rbm4	0.7567	0.0000	7.8056	0.0012	0.0000
Pex7	1.1500	0.0000	7.8092	0.0000	0.0000
Mllt10	0.5100	0.0000	7.8117	0.0000	0.0000
Afg1l	0.8400	0.0000	7.8255	0.0000	0.0000
LOC102639653	0.5100	0.0000	7.8281	0.0001	0.0000
Rit1	1.3433	0.0000	7.8322	0.0028	0.0001
Tfr2	0.5967	0.0000	7.8409	0.0104	0.0002
Ifit1bl2	0.6033	0.0000	7.8534	0.0002	0.0000
Cacnb3	0.6567	0.0000	7.8926	0.0000	0.0000
Polr3e	0.5200	0.0000	7.9252	0.0000	0.0000
Dennd1a	0.4967	0.0000	7.9253	0.0000	0.0000
Rnf144a	0.4067	0.0000	7.9277	0.0000	0.0000
Rpp38	0.8200	0.0000	7.9401	0.0136	0.0003
Btnl10	17.7767	0.0767	7.9461	0.0000	0.0000
Vipas39	0.9067	0.0000	7.9500	0.0000	0.0000
P2ry12	0.8900	0.0000	7.9503	0.0008	0.0000
Wdr25	0.8333	0.0000	7.9578	0.0002	0.0000
Gna15	0.9467	0.0000	7.9608	0.0008	0.0000
Gli2	0.3233	0.0000	7.9635	0.0000	0.0000
Iba57	0.5533	0.0000	7.9637	0.0001	0.0000
Abi2	0.4733	0.0000	7.9657	0.0000	0.0000
Mettl5	2.4533	0.0000	7.9727	0.0000	0.0000
Foxp1	0.3033	0.0000	7.9857	0.0003	0.0000
Invs	0.4067	0.0000	8.0030	0.0003	0.0000
Mcm8	0.7133	0.0000	8.0113	0.0009	0.0000
Nsmf	0.8167	0.0000	8.0249	0.0000	0.0000
Tubb1	9.9433	0.0400	8.0406	0.0022	0.0000
Spns2	0.7967	0.0000	8.0414	0.0005	0.0000
Hivep2	0.2467	0.0000	8.0443	0.0002	0.0000
Gfi1b	14.1567	0.0567	8.0524	0.0280	0.0007
Hars2	0.7900	0.0000	8.0602	0.0015	0.0000
Pms1	0.7900	0.0000	8.0960	0.0000	0.0000
Caprin2	0.8433	0.0000	8.1359	0.0000	0.0000
Ctnnd2	0.4367	0.0000	8.1442	0.0001	0.0000
Klhl15	0.4233	0.0000	8.2082	0.0001	0.0000
Nxpe2	3.0367	0.0167	8.2083	0.0074	0.0002
Epb42	20.0267	0.0700	8.2114	0.0006	0.0000
Clasrp	1.1967	0.0000	8.2154	0.0000	0.0000
Arhgap15	0.9133	0.0000	8.2160	0.0044	0.0001
Gramd1b	0.3600	0.0000	8.2218	0.0000	0.0000
Dact1	0.7133	0.0000	8.2349	0.0000	0.0000
Fancm	0.3600	0.0000	8.2399	0.0000	0.0000
Tanc2	0.2367	0.0000	8.2838	0.0000	0.0000
Dapk2	2.2467	0.0000	8.2898	0.0014	0.0000
Spice1	0.6900	0.0000	8.2934	0.0000	0.0000
Rec8	1.3333	0.0000	8.2983	0.0054	0.0001

Paqr9	16.8667	0.0567	8.3040	0.0163	0.0004
Sirt2	1.3367	0.0000	8.3142	0.0093	0.0002
Gabpb1	1.3900	0.0000	8.3469	0.0070	0.0002
Ccdc66	0.6500	0.0000	8.3664	0.0000	0.0000
Slc2a6	1.4200	0.0000	8.3762	0.0000	0.0000
Gata1	37.8900	0.1167	8.3816	0.0009	0.0000
Zcchc18	1.2200	0.0000	8.3839	0.0265	0.0007
Pdia2	1.7067	0.0000	8.3920	0.0128	0.0003
Rangap1	1.0067	0.0000	8.4110	0.0000	0.0000
Shtn1	0.7467	0.0000	8.4155	0.0001	0.0000
Cd5	1.5067	0.0000	8.4552	0.0000	0.0000
Golga4	0.4400	0.0000	8.5136	0.0000	0.0000
Nt5c3	2.1933	0.0000	8.5213	0.0002	0.0000
Nkx2-5	4.0600	0.0100	8.5259	0.0071	0.0002
Dclk1	0.6600	0.0000	8.5318	0.0000	0.0000
Clstn1	5.2733	0.0200	8.5329	0.0014	0.0000
Sidt1	0.7067	0.0000	8.5333	0.0005	0.0000
Sowaha	4.9300	0.0133	8.5437	0.0002	0.0000
Sh3tc2	0.6933	0.0000	8.5470	0.0000	0.0000
Hbb-bs	30705.2033	83.3300	8.5559	0.0001	0.0000
Ndrg2	1.5933	0.0000	8.5615	0.0000	0.0000
Nr5a1	3.9500	0.0133	8.5945	0.0144	0.0003
Baiap3	0.7067	0.0000	8.5965	0.0001	0.0000
Camp	86.1467	0.2200	8.6064	0.0002	0.0000
Nsg2	1.5567	0.0000	8.6580	0.0436	0.0013
Mllt3	0.6500	0.0000	8.6678	0.0000	0.0000
Psd4	1.1233	0.0000	8.6832	0.0018	0.0000
Sigirr	3.2700	0.0000	8.6891	0.0000	0.0000
Tspan8	2.4567	0.0000	8.7074	0.0001	0.0000
Ddhd2	0.9167	0.0000	8.7327	0.0000	0.0000
Blvrb	5.1233	0.0000	8.7361	0.0001	0.0000
Thap12	1.1133	0.0000	8.7589	0.0000	0.0000
Pfkfb3	0.8033	0.0000	8.7642	0.0015	0.0000
Ptpre	0.6833	0.0000	8.7651	0.0001	0.0000
Miga2	1.1533	0.0000	8.7917	0.0000	0.0000
Tex2	0.8933	0.0000	8.7957	0.0230	0.0006
Hba-a2	29359.9000	67.7933	8.8007	0.0000	0.0000
Gm46223	13.7300	0.0400	8.8073	0.0002	0.0000
Trak1	0.8167	0.0000	8.8697	0.0005	0.0000
Cldn13	75.6133	0.1700	8.8765	0.0038	0.0001
Fam83g	0.8533	0.0000	8.9019	0.0010	0.0000
Hemgn	74.3067	0.1600	8.9056	0.0006	0.0000
Rufy3	1.3133	0.0000	8.9070	0.0001	0.0000
Ccdc88a	1.0200	0.0033	8.9113	0.0432	0.0013
Hba-a1	44420.5333	93.4833	8.9278	0.0000	0.0000
Mlh3	0.7400	0.0000	8.9371	0.0000	0.0000
Nfia	0.8900	0.0033	8.9436	0.0417	0.0012
Odf2	1.2367	0.0000	8.9763	0.0000	0.0000
Prdm1	0.8933	0.0000	9.0174	0.0001	0.0000
Cd1d1	2.3633	0.0000	9.0273	0.0002	0.0000
Tex10	1.7500	0.0000	9.0325	0.0000	0.0000
Rpap1	0.9533	0.0000	9.0521	0.0000	0.0000
Dlg5	0.6500	0.0000	9.0947	0.0000	0.0000
Fam13b	1.0200	0.0000	9.1005	0.0000	0.0000
Atxn2	1.1000	0.0000	9.1260	0.0000	0.0000
Ppm1b	3.2167	0.0067	9.1549	0.0000	0.0000
Zfc3h1	0.7367	0.0000	9.1561	0.0000	0.0000
Ptbp3	0.7567	0.0000	9.1668	0.0078	0.0002
Klf1	87.3967	0.1600	9.1696	0.0000	0.0000
Man1a	1.0800	0.0000	9.1713	0.0000	0.0000
Gp9	12.3667	0.0200	9.1998	0.0032	0.0001
P2ry10b	1.7800	0.0000	9.2459	0.0001	0.0000
Ly6i	5.6133	0.0000	9.2462	0.0000	0.0000
Nacc2	1.6700	0.0033	9.2481	0.0000	0.0000
Map4k4	0.9300	0.0000	9.2772	0.0000	0.0000
Apol11a	23.4733	0.0433	9.2831	0.0009	0.0000
Hbq1b	14.9667	0.0200	9.3173	0.0302	0.0008
Vps8	1.1700	0.0000	9.3469	0.0000	0.0000
Suco	0.9900	0.0000	9.3710	0.0000	0.0000
Tiam1	0.8200	0.0000	9.4318	0.0000	0.0000
Sphk1	3.9900	0.0000	9.4330	0.0000	0.0000
Trim58	5.5000	0.0067	9.4679	0.0270	0.0007
Agpat1	3.3467	0.0000	9.4818	0.0000	0.0000
Osbpl3	0.9733	0.0000	9.5093	0.0001	0.0000
Crispld2	1.5800	0.0000	9.5133	0.0000	0.0000
Prr14l	0.6233	0.0000	9.5149	0.0000	0.0000
Rnf216	1.4567	0.0000	9.5342	0.0000	0.0000
Tfdp2	0.9233	0.0000	9.5599	0.0002	0.0000
Trp53i11	2.1733	0.0000	9.5616	0.0000	0.0000
Nkx2-3	6.0167	0.0067	9.5748	0.0242	0.0006
Mapk7	2.3867	0.0000	9.6025	0.0000	0.0000
Cramp1l	0.9167	0.0000	9.6278	0.0000	0.0000

Rtn4rl1	2.0900	0.0000	9.6424	0.0000	0.0000
Rps6kb1	1.5400	0.0000	9.6730	0.0000	0.0000
1300017J02Rik	6.1733	0.0067	9.6934	0.0008	0.0000
Tent5a	1.3367	0.0000	9.7074	0.0000	0.0000
Serping1	3.9333	0.0000	9.7130	0.0000	0.0000
Acp5	2.9100	0.0000	9.7716	0.0012	0.0000
Ubtf	1.8067	0.0000	9.7877	0.0000	0.0000
Papola	1.7867	0.0033	9.8019	0.0141	0.0003
Csnk1a1	1.8700	0.0000	9.8092	0.0002	0.0000
Cflar	1.2067	0.0000	9.8258	0.0000	0.0000
Zmiz2	1.7067	0.0000	9.8297	0.0000	0.0000
Hbb-bt	5994.2867	6.8433	9.8308	0.0000	0.0000
Abcc9	1.0633	0.0000	9.8482	0.0000	0.0000
Zfp180	2.2567	0.0000	9.9299	0.0000	0.0000
Clec1b	7.3767	0.0000	9.9339	0.0009	0.0000
Tcf21	13.8200	0.0133	9.9725	0.0168	0.0004
Ngp	243.7567	0.2533	9.9769	0.0000	0.0000
Hmbs	192.1033	0.2100	9.9934	0.0016	0.0000
Tlx1	8.6267	0.0067	10.0369	0.0163	0.0004
Gnptab	1.9700	0.0000	10.0408	0.0000	0.0000
Spta1	11.7633	0.0100	10.0519	0.0023	0.0000
Sh2b3	2.3933	0.0000	10.0912	0.0000	0.0000
Elane	37.5433	0.0333	10.1248	0.0025	0.0001
Itpr2	0.8433	0.0000	10.1369	0.0000	0.0000
Sox6	1.2100	0.0000	10.2152	0.0015	0.0000
Lpin2	1.8500	0.0000	10.2498	0.0000	0.0000
Mpo	30.6700	0.0267	10.2914	0.0000	0.0000
Cbfa2t3	1.6700	0.0000	10.4866	0.0001	0.0000
Myo7a	1.8233	0.0000	10.5543	0.0000	0.0000
Golim4	3.3600	0.0000	10.7375	0.0000	0.0000
Brd4	2.6633	0.0000	10.8251	0.0000	0.0000
Apol10a	10.2233	0.0067	10.8309	0.0082	0.0002
Apol11b	173.2600	0.1000	10.8863	0.0000	0.0000
Rhag	38.2933	0.0267	10.8900	0.0143	0.0003
Mtmr12	3.8967	0.0000	10.8935	0.0000	0.0000
Ipcef1	2.7467	0.0000	10.9372	0.0000	0.0000
Sfswap	5.2433	0.0000	10.9549	0.0489	0.0014
Sdcbp	6.8067	0.0000	10.9779	0.0482	0.0014
Trim10	41.9767	0.0200	10.9910	0.0009	0.0000
Gdpd2	13.7067	0.0067	10.9918	0.0068	0.0001
Ifit2	4.5233	0.0000	11.0026	0.0482	0.0014
Slc25a38	10.3800	0.0000	11.0159	0.0002	0.0000
Btrc	4.0300	0.0000	11.0486	0.0467	0.0014
Slc43a1	7.7600	0.0000	11.0495	0.0460	0.0013
Fcrla	10.9533	0.0000	11.0498	0.0460	0.0013
Kmt2d	1.0167	0.0000	11.0530	0.0023	0.0000
Apol8	6.7133	0.0000	11.0749	0.0457	0.0013
Treml4	11.3300	0.0000	11.1636	0.0430	0.0012
Specc1	3.3100	0.0000	11.1945	0.0433	0.0013
Pklr	7.7900	0.0000	11.2911	0.0395	0.0011
Slc4a1	283.7067	0.1133	11.3513	0.0000	0.0000
Kel	37.1867	0.0133	11.4808	0.0046	0.0001
Tbcel	5.5700	0.0000	11.5550	0.0000	0.0000
Car2	15.1467	0.0000	11.6076	0.0320	0.0009
Pik3r1	4.7633	0.0000	11.6342	0.0000	0.0000
Slfn14	7.5433	0.0000	11.6447	0.0328	0.0009
Tspo2	36.5733	0.0000	11.9162	0.0261	0.0007
Gramd3	13.2933	0.0000	11.9367	0.0260	0.0007
Gm1966	5.0933	0.0000	12.3173	0.0001	0.0000
Fnbp1	8.3300	0.0000	12.3451	0.0204	0.0005
Bnip3l	14.5600	0.0000	12.3551	0.0000	0.0000
Slc38a5	25.2733	0.0000	12.4056	0.0189	0.0005
Cpeb4	6.5233	0.0000	12.4601	0.0000	0.0000
Ppp1r18	17.8867	0.0000	12.4763	0.0204	0.0005
Gypa	114.1967	0.0167	12.6917	0.0023	0.0000
Arrb1	8.5367	0.0000	12.7694	0.0000	0.0000
Add2	7.6100	0.0000	12.7844	0.0150	0.0004
Lmo2	42.3000	0.0000	12.8371	0.0147	0.0004
Car1	84.7133	0.0000	13.4833	0.0092	0.0002
