# Supplementary material for: Identification of CBPA as a New Inhibitor of PD-1/PD-L1 Interaction
Source: Int J Mol Sci. 2023 Feb 16;24(4):3971. doi: 10.3390/ijms24043971 (PMC9964281; doi:10.3390/ijms24043971)
Supplement: Supplementary file 1 [file ijms-24-03971-s001.zip › supplementary/supplementary Table S3.pdf]

Table S3. KEGG pathway terms enriched through GSEA

Enriched KEGG pathway terms by genes down-expressed in CBPA-treated group

KEGG Pathway Term	NES	FDR q-val
Ribosome	-2.2520	<0.0001
Carbon metabolism	-2.0960	7.01E-04
Carbon fixation in photosynthetic organisms	-2.0461	1.36E-03
Biosynthesis of antibiotics	-2.0645	1.37E-03
Biosynthesis of amino acids	-1.9305	8.89E-03
Methane metabolism	-1.9475	9.25E-03
Citrate cycle (TCA cycle)	-1.8932	1.20E-02
Relaxin signaling pathway	-1.8212	2.21E-02
Focal adhesion	-1.7799	3.06E-02
Vasopressin-regulated water reabsorption	-1.7644	3.14E-02
Glycolysis / Gluconeogenesis	-1.7515	3.16E-02
Fructose and mannose metabolism	-1.6870	4.68E-02
ECM-receptor interaction	-1.6926	4.80E-02

Enriched KEGG pathway terms by genes up-expressed in CBPA-treated group

KEGG Pathway Term	NES	FDR q-val
Hematopoietic cell lineage	2.7267	<0.0001
Intestinal immune network for IgA production	2.4479	<0.0001
Antigen processing and presentation	2.3094	<0.0001
Natural killer cell mediated cytotoxicity	2.2978	<0.0001
Th1 and Th2 cell differentiation	2.1721	<0.0001
Cytokine-cytokine receptor interaction	2.1705	<0.0001
Cell adhesion molecules (CAMs)	2.1693	<0.0001
NOD-like receptor signaling pathway	2.0240	8.81E-04
ABC transporters	2.0262	8.95E-04
NF-kappa B signaling pathway	2.0104	1.00E-03
Th17 cell differentiation	1.9979	1.11E-03
T cell receptor signaling pathway	1.9860	1.19E-03
Jak-STAT signaling pathway	1.9200	2.80E-03
B cell receptor signaling pathway	1.9100	2.89E-03
Osteoclast differentiation	1.8894	3.16E-03
Neuroactive ligand-receptor interaction	1.8695	3.49E-03
Cytosolic DNA-sensing pathway	1.8045	7.36E-03
Platelet activation	1.7511	1.22E-02
Fc epsilon RI signaling pathway	1.7343	1.37E-02
Necroptosis	1.7265	1.41E-02
RIG-I-like receptor signaling pathway	1.6282	3.33E-02
Phagosome	1.6030	3.92E-02
