# Supplementary figures and images for: Identification of CBPA as a New Inhibitor of PD-1/PD-L1 Interaction
Source: Int J Mol Sci. 2023 Feb 16;24(4):3971. doi: 10.3390/ijms24043971 (PMC9964281; doi:10.3390/ijms24043971)

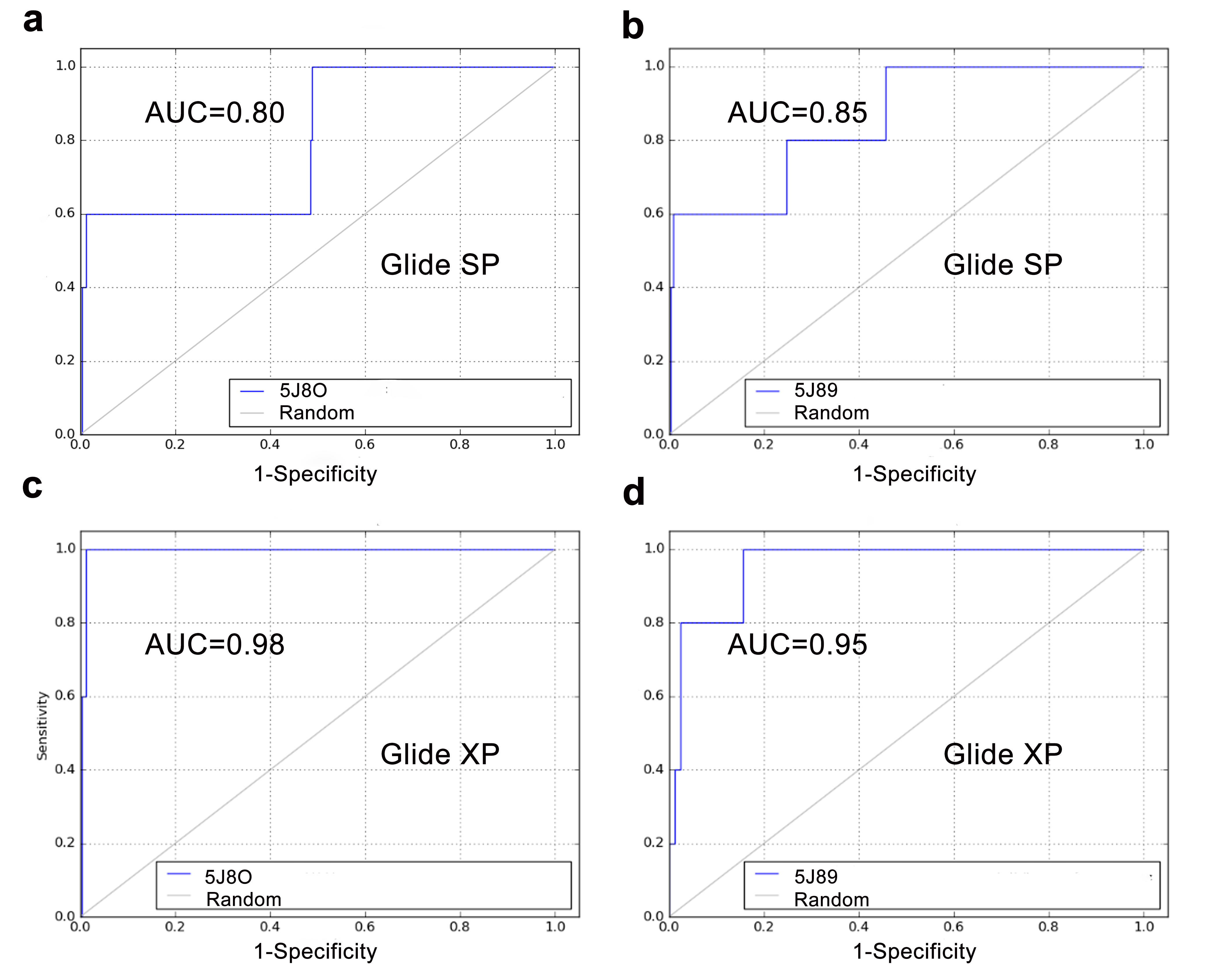

Supplement: Supplementary file 1 [file ijms-24-03971-s001.zip › supplementary/FigS1.tif]

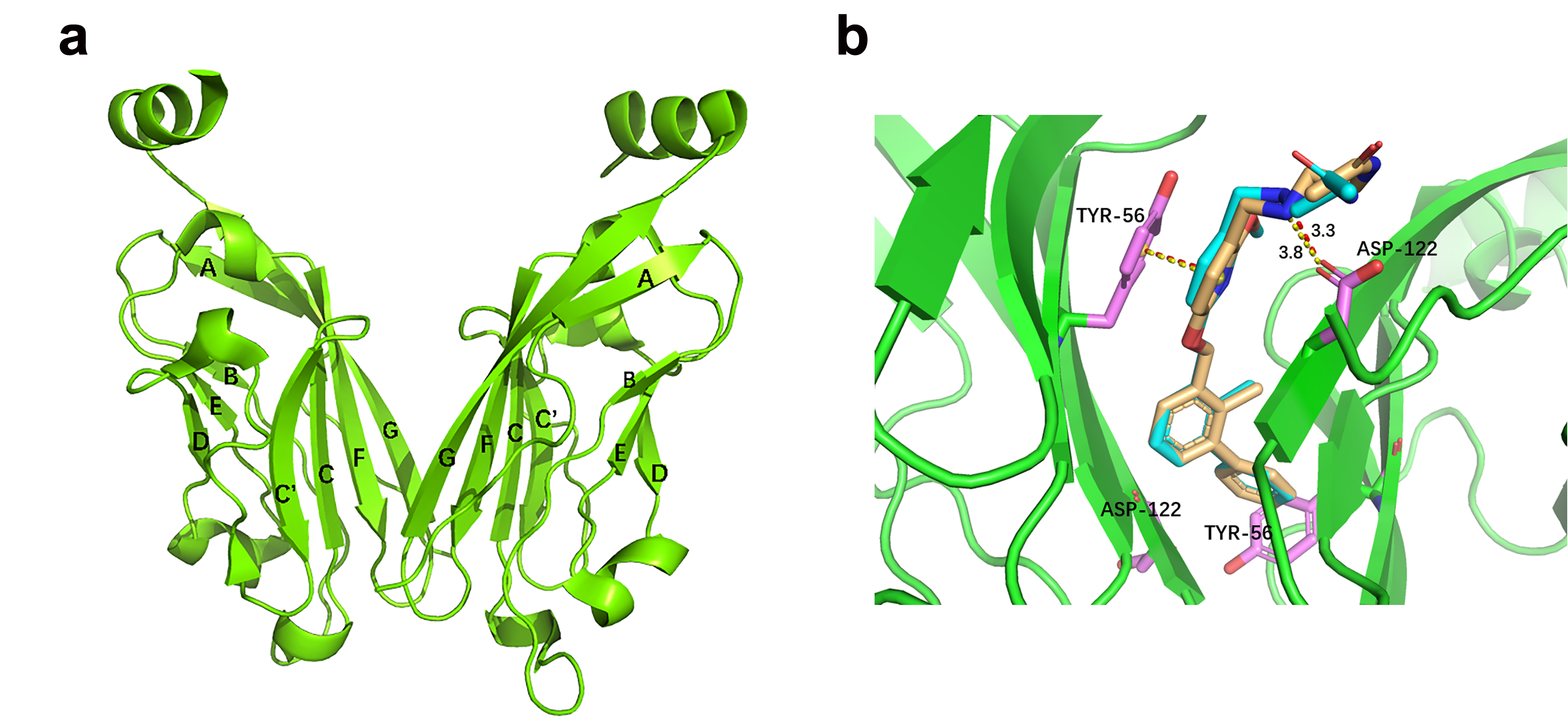

Supplement: Supplementary file 1 [file ijms-24-03971-s001.zip › supplementary/FigS2.tif]

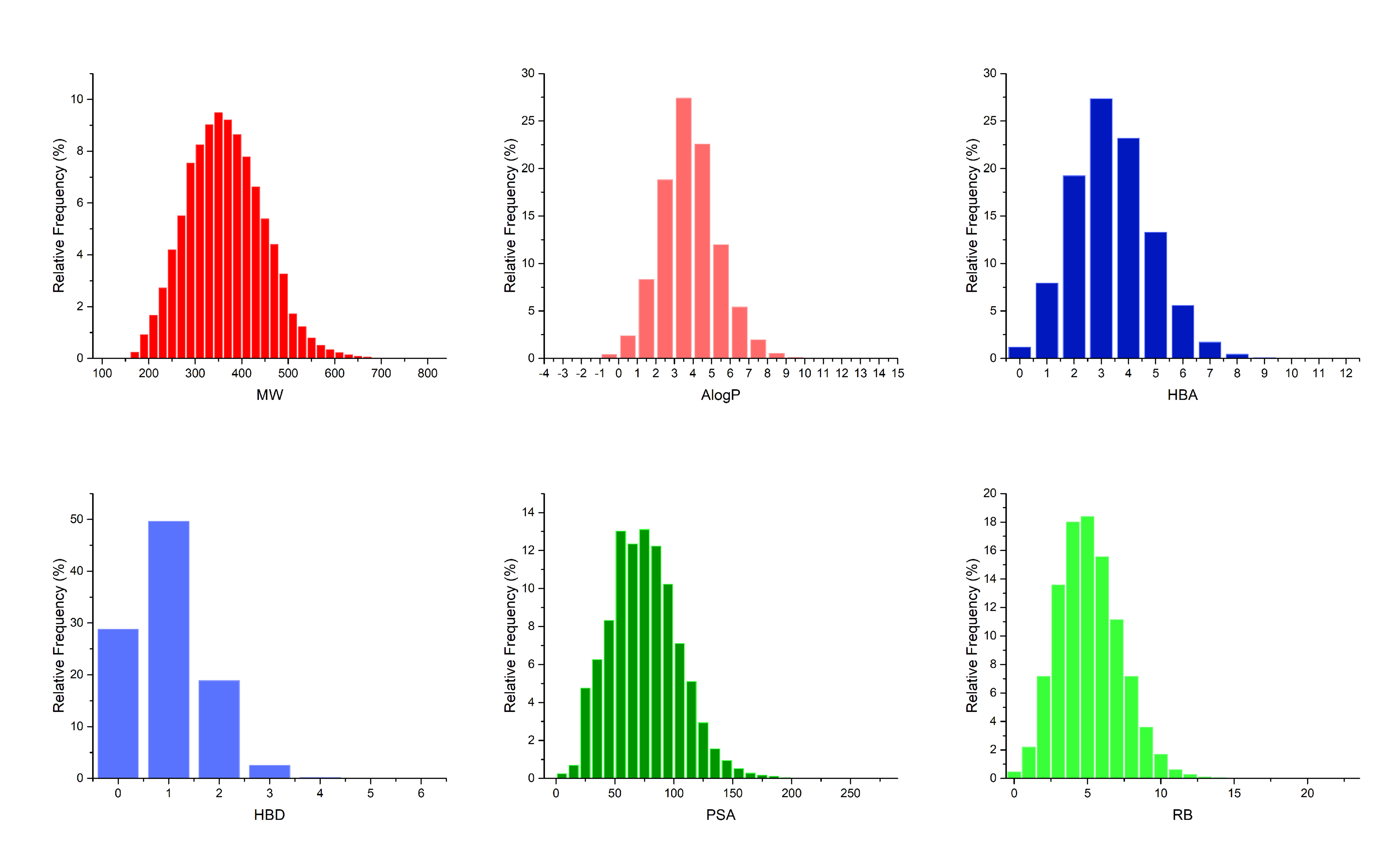

Supplement: Supplementary file 1 [file ijms-24-03971-s001.zip › supplementary/FigS3.tif]

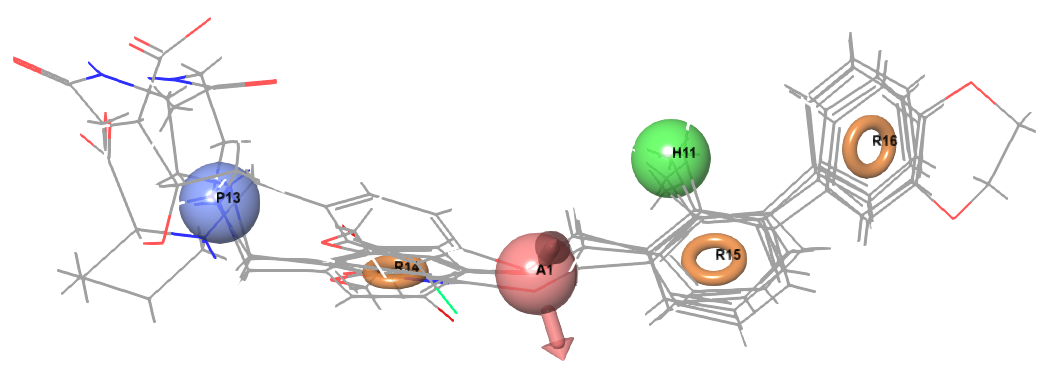

Supplement: Supplementary file 1 [file ijms-24-03971-s001.zip › supplementary/FigS4.png]

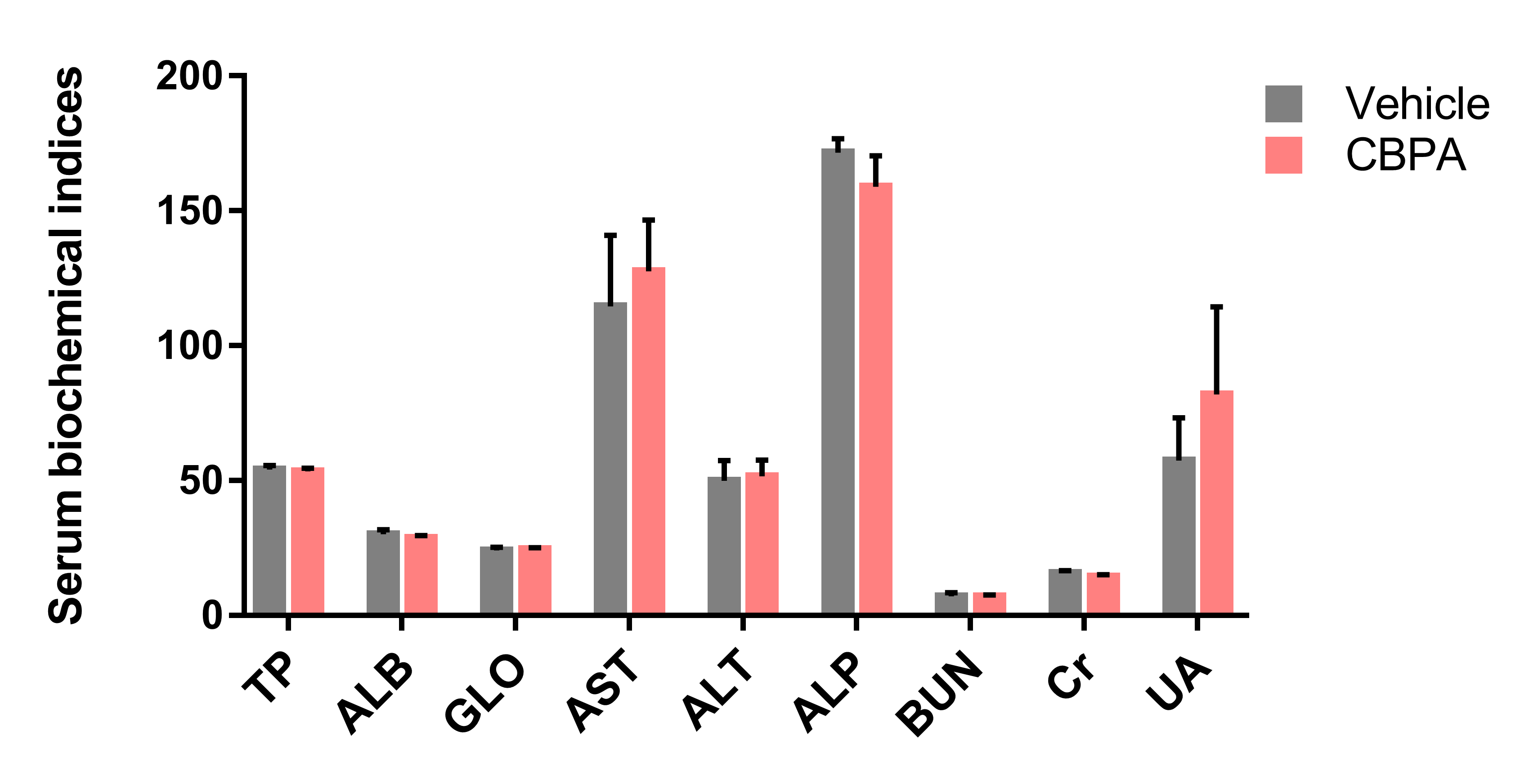

Supplement: Supplementary file 1 [file ijms-24-03971-s001.zip › supplementary/FigS5.tif]

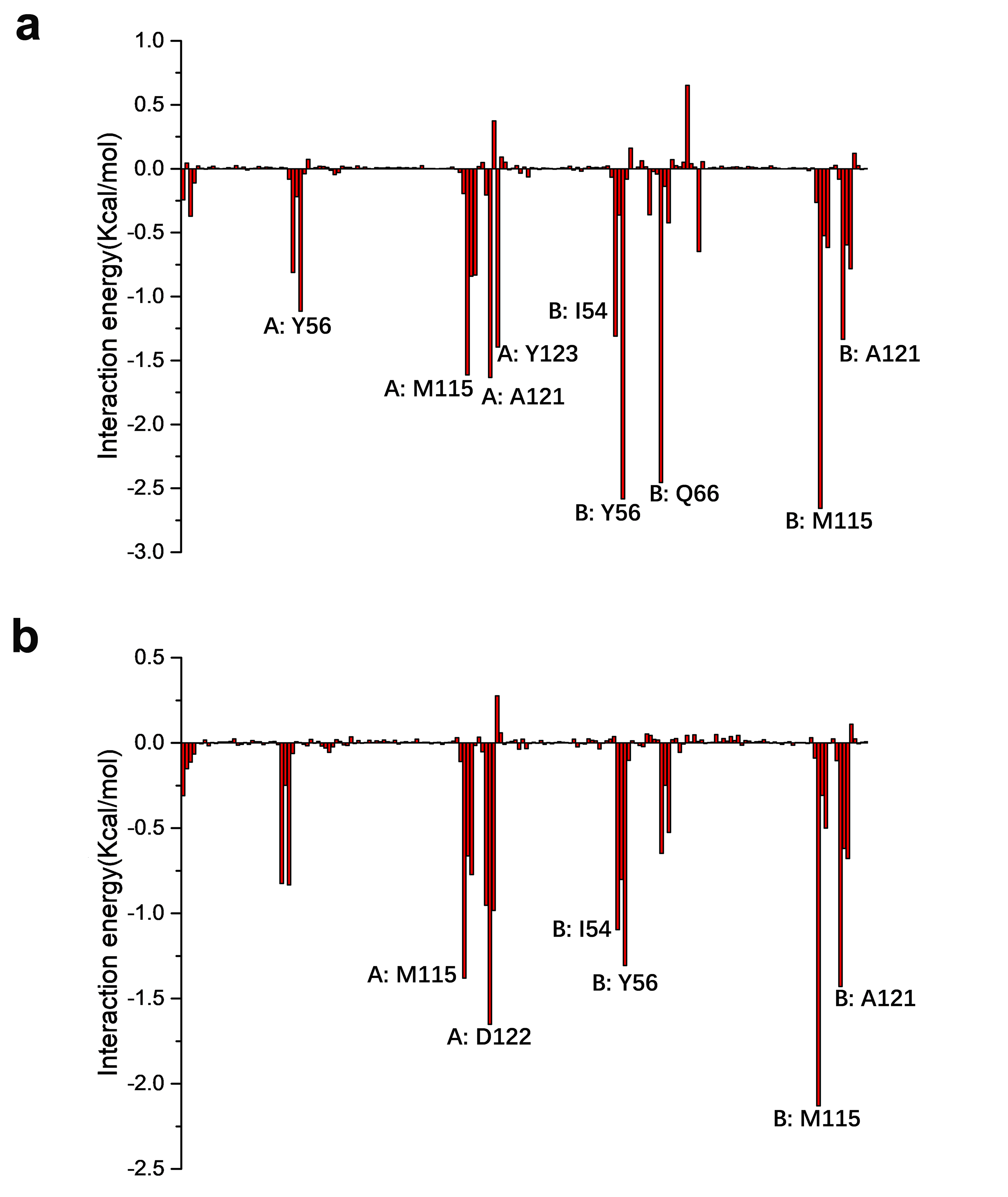

Supplement: Supplementary file 1 [file ijms-24-03971-s001.zip › supplementary/FigS6.tif]

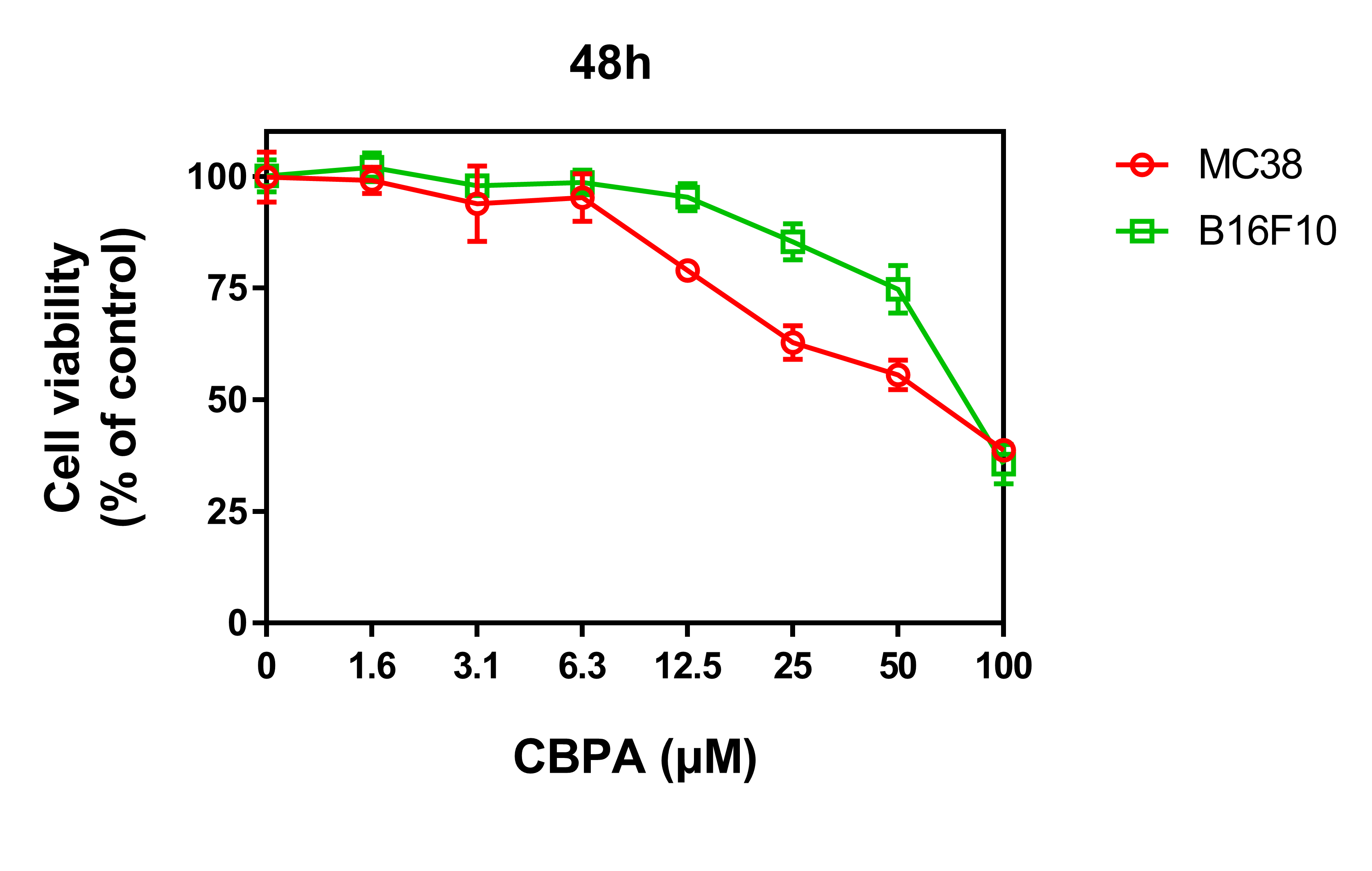

Supplement: Supplementary file 1 [file ijms-24-03971-s001.zip › supplementary/FigS7.tif]
